# Mutations in *GRHL2* Result in an Autosomal-Recessive Ectodermal Dysplasia Syndrome

**DOI:** 10.1016/j.ajhg.2014.08.001

**Published:** 2014-09-04

**Authors:** Gabriela Petrof, Arti Nanda, Jake Howden, Takuya Takeichi, James R. McMillan, Sophia Aristodemou, Linda Ozoemena, Lu Liu, Andrew P. South, Celine Pourreyron, Dimitra Dafou, Laura E. Proudfoot, Hejab Al-Ajmi, Masashi Akiyama, W.H. Irwin McLean, Michael A. Simpson, Maddy Parsons, John A. McGrath

**Affiliations:** 1 St. John’s Institute of Dermatology, King’s College London, Guy’s Campus, London SE1 9RT, UK; 2 As’ad Al-Hamad Dermatology Center, Al-Sabah Hospital, Kuwait City 13001, Kuwait; 3 Randall Division of Cell and Molecular Biophysics, King’s College London, Guy’s Campus, London SE1 9RT, UK; 4 Department of Dermatology, Nagoya University Graduate School of Medicine, Nagoya 466-8560, Japan; 5 National Diagnostic Epidermolysis Bullosa Laboratory, Viapath, St. Thomas’ Hospital, London SE1 7EH, UK; 6 Dermatology and Genetic Medicine, College of Life Sciences and College of Medicine, Dentistry, and Nursing, University of Dundee, Dundee DD1 5EH, UK; 7 Department of Genetics, Development, and Molecular Biology, School of Biology, Aristotle University, Thessaloniki 54124, Greece; 8 Department of Medical and Molecular Genetics, King’s College London School of Medicine and Guy’s Hospital, London SE1 9RT, UK

## Abstract

Grainyhead-like 2, encoded by *GRHL2*, is a member of a highly conserved family of transcription factors that play essential roles during epithelial development. Haploinsufficiency for *GRHL2* has been implicated in autosomal-dominant deafness, but mutations have not yet been associated with any skin pathology. We investigated two unrelated Kuwaiti families in which a total of six individuals have had lifelong ectodermal defects. The clinical features comprised nail dystrophy or nail loss, marginal palmoplantar keratoderma, hypodontia, enamel hypoplasia, oral hyperpigmentation, and dysphagia. In addition, three individuals had sensorineural deafness, and three had bronchial asthma. Taken together, the features were consistent with an unusual autosomal-recessive ectodermal dysplasia syndrome. Because of consanguinity in both families, we used whole-exome sequencing to search for novel homozygous DNA variants and found *GRHL2* mutations common to both families: affected subjects in one family were homozygous for c.1192T>C (p.Tyr398His) in exon 9, and subjects in the other family were homozygous for c.1445T>A (p.Ile482Lys) in exon 11. Immortalized keratinocytes (p.Ile482Lys) showed altered cell morphology, impaired tight junctions, adhesion defects, and cytoplasmic translocation of GRHL2. Whole-skin transcriptomic analysis (p.Ile482Lys) disclosed changes in genes implicated in networks of cell-cell and cell-matrix adhesion. Our clinical findings of an autosomal-recessive ectodermal dysplasia syndrome provide insight into the role of GRHL2 in skin development, homeostasis, and human disease.

## Main Text

Grainyhead-like 2 (GRHL2) is a mammalian homolog of *Drosophila* protein grainy head (GRH), which, along with GRHL1 and GRHL3, has a role in epithelial morphogenesis.^1,2^ This family of transcription factors controls the development and differentiation of multicellular epithelia by regulating genes germane to cell junction formation and proliferation.^3,4^ Biologically, GRHL2 contributes to formation of the epithelial barrier and wound healing, as well as neural-tube closure, maintenance of the mucociliary airway epithelium, and tumor suppression.^5–11^ *GRHL2* (MIM 608576) has been shown to regulate *TERT* (MIM 187270) expression and to enhance proliferation of epidermal keratinocytes; it also impairs keratinocyte differentiation through transcription inhibition of genes clustered at the epidermal differentiation complex^12^ and regulates epithelial morphogenesis by establishing functional tight junctions.^13^

GRHL2 is also present in the cochlear duct,^14^ and mutations in human *GRHL2* have been found in progressive autosomal-dominant hearing loss (DFNA28 [MIM 608641]),^15,16^ and other polymorphic sequence variants in *GRHL2* have been implicated in age-related hearing impairment and noise-induced hearing loss.^17–19^ To date, however, the role of GRHL2 in skin biology has not been well established. Causing severe facial and neural-tube defects, *Grhl2* knockout is embryonically lethal in mice,^17,20^ and mutant zebrafish display inner-ear defects and abnormal swimming positions.^18^ In contrast, *Grhl1*^*−/−*^ mice show hair loss and palmoplantar keratoderma, as well as abnormal desmosome cell junctions and dysregulated terminal differentiation in keratinocytes.^21^ Moreover, *Grhl3*^*−/−*^ embryos fail to establish a normal epidermal barrier and display defective embryonic wound repair.^22^ Thus, unlike for GRHL1 and GRHL3, there is currently a lack of data associating GRHL2 with skin pathology. In this report, however, we have identified two families in which affected subjects have developmental defects affecting skin, oral mucosa, and teeth (as well as hearing and lungs), thus implicating *GRHL2* in an autosomal-recessive ectodermal dysplasia syndrome.

**Figure 1:**
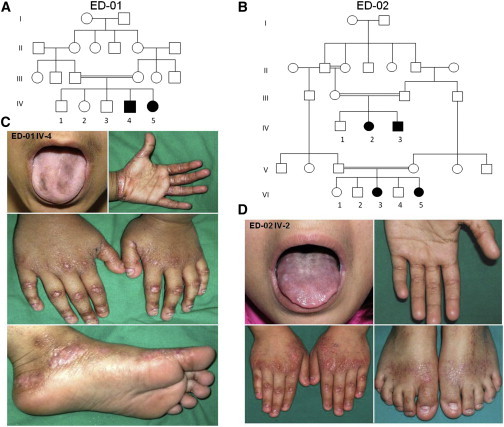
Pedigrees and Clinical Features of This Autosomal-Recessive Ectodermal Dysplasia Syndrome (A and B) Two unrelated consanguineous pedigrees with a total of six affected individuals. (C and D) An affected 8-year-old male (from pedigree ED-01) and an affected 12-year-old female (from pedigree ED-02) both show features of tongue hyperpigmentation, skin thickening around the margins of the palms and soles, hypoplastic finger and toe nails, knuckle pads on the fingers, and atrophic wrinkling on the dorsal aspects of the hands and feet. Additional clinical images from other subjects are shown in Figure S1.

We investigated two unrelated Kuwaiti families, both consanguineous, in which clinically similar features were present in a total of six affected individuals (Figures 1A and 1B). The clinical features were noted in early infancy and comprised short stature (≤25^th^ percentile), nail dystrophy and/or loss, oral mucosa and/or tongue pigmentation, abnormal dentition (delay, hypodontia, enamel hypoplasia), keratoderma affecting the margins of the palms and soles, and focal hyperkeratosis of the dorsal aspects of the hands and feet (Figures 1C and 1D; Figure S1, available online). No individual showed any wound-healing defect, blistering tendency, hair or sweating abnormalities, or other developmental anomalies. Two affected sisters (ED-02 VI-3 and VI-5) had dysphagia with evident esophageal strictures. Three individuals (ED-01 IV-4 and IV-5 and ED-02 VI-3) developed sensorineural deafness in early infancy, and three others (ED-01 IV-4 and IV-5 and ED-02 IV-2) had bronchial asthma. One individual (ED-01 IV-4) had severe iron-deficiency anemia requiring blood transfusion. Laboratory tests (full blood count, serum biochemistry, immunoglobulin levels, and thyroid-function tests) were otherwise within the normal range for all affected individuals. None of the parents had any skin, hair, teeth, nail, or hearing abnormalities.

To investigate the etiology of the condition, we first assessed lesional skin biopsies taken from three affected individuals (ED-01 IV-4 and ED-02 VI-3 and VI-5) by using immunohistochemistry and transmission electron microscopy. The subjects’ legal guardians provided written informed consent according to a protocol approved by the St. Thomas’ Hospital Ethics Committee (Molecular basis of inherited skin disease: 07/H0802/104). Blood and skin samples (ellipse of skin taken under local anesthesia by 1% lignocaine) were obtained in adherence to the Declaration of Helsinki guidelines. Light microscopy showed mild acanthosis and hyperkeratosis (Figure S2), but transmission electron microscopy of the skin was unremarkable—it showed no clear abnormalities in keratinocytes, hemidesmosomes, or desmosome cell-cell junctions. Likewise, skin immunolabeling using a panel of antibodies that target basement membrane extracellular matrix proteins (collagen IV and collagen VII), epidermal-adhesion-associated proteins (desmoglein 1, keratin 1, keratin 14, and desmoplakin), and markers of terminal differentiation (filaggrin) showed normal intensity and labeling patterns when affected skin from two affected individuals was compared to skin from a normal control subject (Figure S3; see Supplemental Data for methods and antibody details). However, we noted increased staining for the proliferation marker Ki-67 in the epidermis (Figure S4). Collectively, the skin-biopsy findings were not diagnostic for any known inherited skin disease.

**Figure 2:**
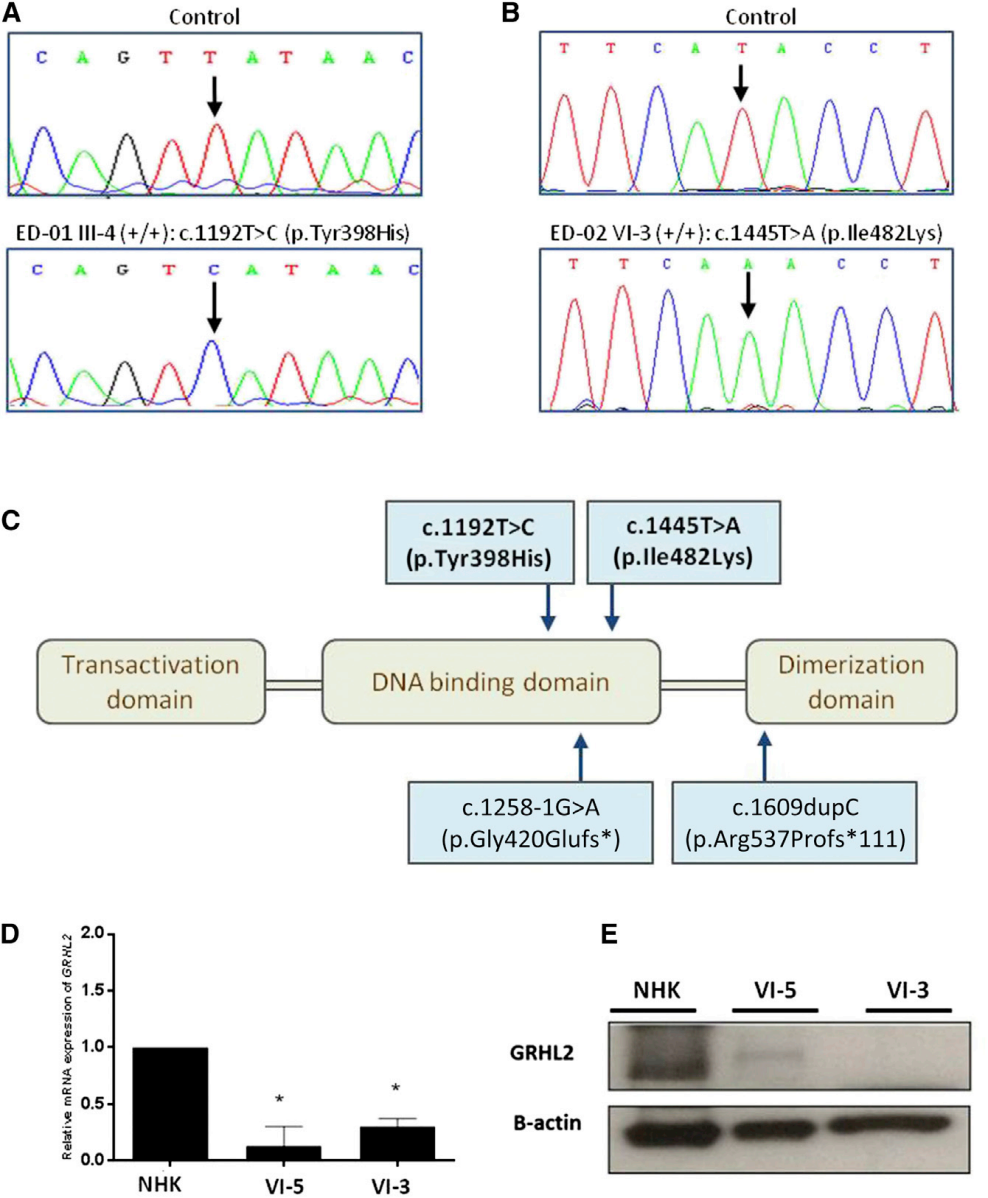
Autosomal-Recessive Mutations in *GRHL2* Lead to Reduced Gene Expression and Protein Levels (A and B) Sanger sequencing confirmed the presence of different homozygous missense mutations in *GRHL2* in affected subjects from both pedigrees. (C) Schematic representation of the functional domains of GRHL2. The recessive missense mutations we identified (top) are located within the DNA binding domain; the previously reported heterozygous splice-site or deletion mutations that cause autosomal-dominant deafness are also illustrated (bottom). (D) qPCR for *GRHL2* expression in cultured keratinocytes showed reduced expression in two affected subjects from pedigree ED-02 (^∗^p < 0.05 in comparison to control cells). Error bars represent the SD from three independent experiments. (E) Immunoblotting using cultured keratinocyte whole-cell lysates revealed markedly reduced or undetectable amounts of GRHL2 in these same individuals.

We then used whole-exome sequencing to identify a candidate gene or genes. We extracted genomic DNA from peripheral blood from two affected individuals (ED-01 IV-4 and ED-02 VI-3). We performed whole-exome capture by using in-solution hybridization (Agilent All Exon Kit V4) and generated sequencing on the Illumina HiSeq 2000. Resulting reads were aligned to the reference human genome (UCSC Genome Browser hg19, GRCh37) with the Novoalign software package (Novocraft Technologies). Duplicate reads, resulting from PCR clonality or optical duplicates, and reads mapping to multiple locations were excluded from downstream analysis. A summary of exome-coverage data is presented in Table S1. Sixteen previously unreported homozygous mutations were identified (ten in family ED-01 and six in family ED-02). The only gene containing homozygous variants common to both subjects was *GRHL2* (Table S2). The respective mutations were c.1192T>C (p.Tyr398His) in exon 9 and c.1445T>A (p.Ile482Lys) (RefSeq NM_024915.3) in exon 11. These mutations were confirmed by Sanger sequencing (Figures 2A and 2B) and were also shown to segregate with the disease phenotype in other affected pedigree members. Both mutations are located in the DNA binding site of *GRHL2* (Figure 2C) and are predicted to be “probably damaging” by PolyPhen-2 analysis (scores 0.984 and 0.994 for c.1192T>C and c.1445T>A, respectively). Neither variant has been observed by the 1000 Genomes Project or detected in ∼1,200 control in-house exomes or in 260 ethnically matched control chromosomes.

**Figure 3:**
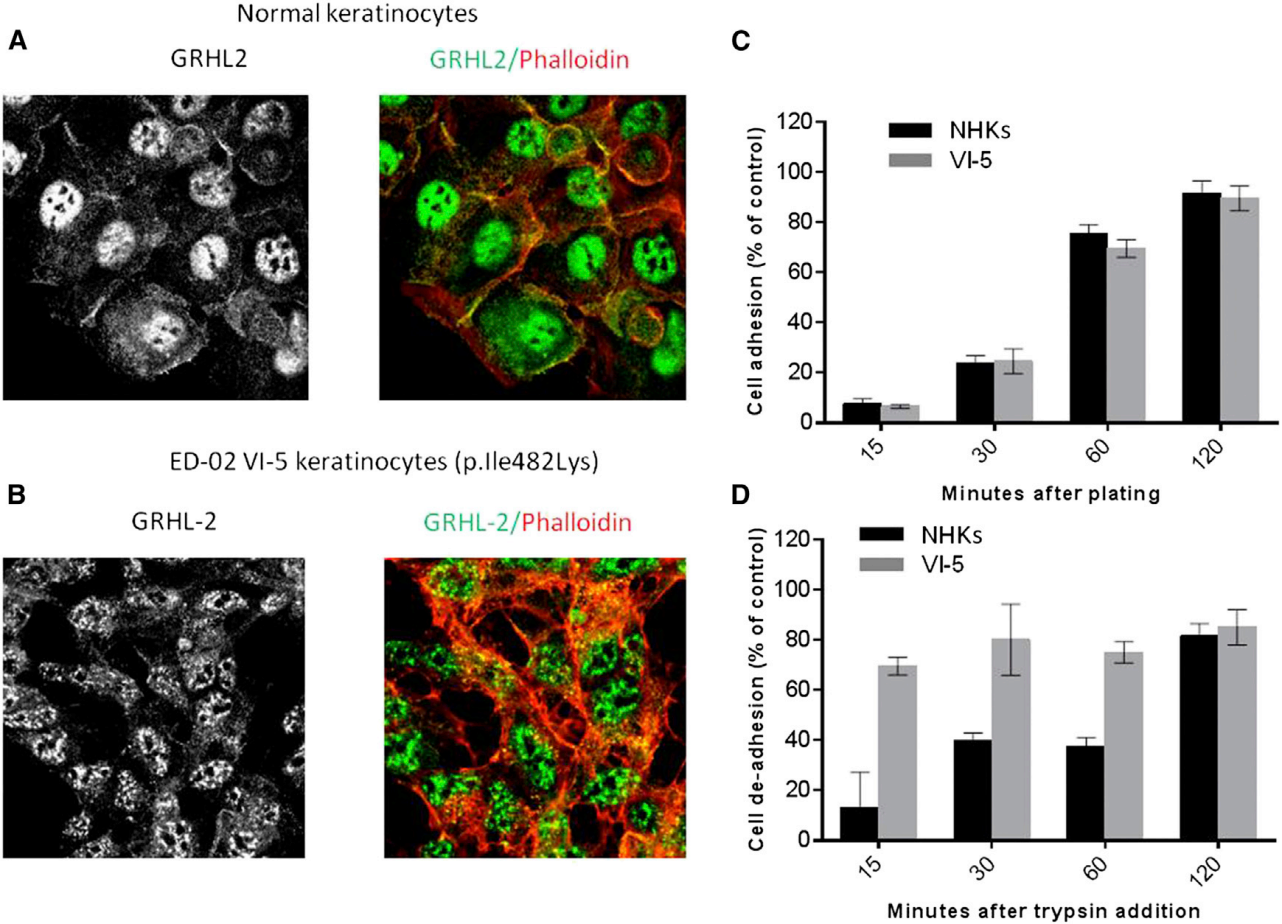
Impact of *GRHL2* Mutations on Keratinocyte Cell Biology (A) Confocal microscopy in normal keratinocytes revealed nuclear, cytoplasmic, and membranous labeling for an antibody raised against GRHL2. (B) In contrast, keratinocytes from an affected subject showed an altered pattern of antibody localization within the nucleus and a lack of any cell membrane labeling. (C) Cell-adhesion assays showed no difference between wild-type and mutant keratinocytes. Error bars represent the SD from three independent experiments. (D) In contrast, mutant cells showed more rapid detachment in trypsin de-adhesion assays. Error bars represent the SD from three independent experiments. NHK stands for normal human keratinocyte.

GRHL2 can be detected in the nuclear fraction of normal human keratinocytes in culture (but is subsequently lost in cell senescence).^23^ We cultured keratinocytes from two of the skin biopsies (ED-02 VI-3 and ED-02 VI-5) by standard methods and used these cells to examine *GRHL2* expression and GRHL2 localization (see Supplemental Data for methods); both were found to be reduced (Figures 2D and 2E). We then isolated primary keratinocytes from one of the affected individuals (ED-02 VI-5) and immortalized these cells at passage 1 (see Supplemental Data for methods). The phenotype of these cells was assessed by confocal microscopy. *GRHL2* mutant cells showed a less cuboidal, elongated phenotype and failed to form intact cell junctions, as seen in control immortalized keratinocytes. Notably, there was a reduction in cell membrane labeling for E-cadherin (adherens junctions) and zona-occludens-2 (tight junctions) (Figure S5). GRHL2 staining in control keratinocytes was seen both at cell-cell contact areas and within the nucleus (Figure 3A), whereas in mutant cells, the signal was not at the periphery and instead showed a fragmented punctate nuclear localization (Figure 3B). To assess the effects of the mutations on keratinocyte cell function, we also performed assays of cell adhesion and de-adhesion (see Supplemental Data for methods). No differences were noted for cell adhesion between mutant and control cells (Figure 3C), but mutant cells detached from fibronectin much faster than normal human keratinocyte controls after exposure to trypsin (Figure 3D).

Next, we assessed the transcriptome profile by using RNA extracted from whole skin from two individuals in pedigree ED-02. RNA from healthy control skin was obtained from discarded abdominoplasty tissue from plastic surgeons and used as four pooled samples. RNA extraction was performed with the Ambion mirVana miRNA Isolation kit (Invitrogen) according to the manufacturer’s instructions. RNA was amplified with the Illumina TotalPrep RNA Amplification Kit, and subsequent gene-expression profiling was performed with the Illumina array HumanHT-12 v4 Expression BeadChip Kit according to the manufacturer’s instructions. Gene-expression data were then analyzed with GenomeStudio software (Illumina). A prefiltering set was determined for significantly modulated expression (detection p value < 0.01; signal intensity fold change ≥ 2.0) between affected and control skin. A comprehensive functional-enrichment analysis was then performed with (1) the Database for Annotation, Visualization, and Integrated Discovery (v.6.7), based on the Gene Ontology (GO) database (see Web Resources), and (2) the GeneGo Metacore software (Thomson Reuters), a systems-biology analysis tool based on a curated database of human protein-protein and protein-DNA interactions, transcription factors, signaling, and metabolic pathways. Comparison of the affected individuals’ skin with the skin of healthy age- and site-matched control individuals identified 1,457 gene transcripts that were significantly altered: 668 upregulated (≥2-fold change) and 789 downregulated (≤0.5-fold change) transcripts for ED-02 VI-5. For ED-02 VI-3, 1,141 gene transcripts were altered: 466 upregulated and 675 downregulated. Of these changes in gene expression, 359 upregulated and 344 downregulated gene transcripts were common to both affected subjects. Evaluation of the changes in gene expression by functional-enrichment analysis identified several enriched GO pathways, processes, networks, and disease-associated transcripts, some of which are germane to the known functions of GRHL2. The top three upregulated GO pathways were linked to protein-folding maturation, cytoskeleton remodeling, and transcriptional control of lipid biosynthesis (involving genes encoding proopiomelanocortins and mitochondrial enzymes involved in metabolic pathways) (Tables S3–S10). Among the most significantly upregulated GO networks were the signal-transduction pathways and intermediate-filament remodeling (Table S7). Conversely, immune-response signaling; migration-inhibitory-factor-induced cell adhesion, migration, and angiogenesis; and networks of cell-cell and cell-matrix adhesion were downregulated (Table S8).

With regard to skin differentiation and barrier formation, selected alterations in gene expression are presented in Table S11. We also verified potential changes by performing quantitative PCR (qPCR) with RNA from whole skin of three individuals from the two pedigrees, as well as immortalized keratinocytes and primary fibroblasts from one affected person (see Supplemental Data for methods and controls). We observed reduced expression of *GRHL2* for all templates and a contrasting increase in *GRHL1* (MIM 609786) and *GRHL3* (MIM 608317) expression: unique and cooperative roles for this transcription factor family have been previously documented.^24^ The most marked skin-barrier-associated gene changes were upregulation of aquaporin-encoding genes *AQP5* (MIM 600442) and *AQP7* (MIM 602974), the latter of which was expressed 50×–100× more in affected skin than in control skin. Gain-of-function mutations in *AQP5* have previously been associated with a form of autosomal-dominant nonepidermolytic palmoplantar keratoderma (MIM 600962).^25^ Two-fold or greater reduction in gene expression was noted for *S100A8* (MIM 123885) and *S100A9* (MIM 123886), known targets for *GRHL1*. Previously, it has also been shown that GRHL2 enhances skin-barrier function by upregulating the tight-junction components claudins 3 and 4 and also Rab25, which localizes claudin 4 to tight junctions.^26^ In affected people, we noted increases in *CLDN3* (MIM 602910), *CLDN4* (MIM 602909), and *RAB25* (MIM 612942) expression in whole skin (transcriptome and qPCR) and cultured keratinocytes (qPCR). Increased claudin 4 immunolabeling was also noted in the skin of two affected individuals (Figure S5).

Labeling for the proliferation marker Ki-67 was increased in the affected subjects’ skin (Figure S4). This indicates that suprabasal keratinocytes are subject to an abnormal terminal-differentiation program, which provides a possible explanation for thickening of the epidermis and impairment of the epidermal barrier in our affected individuals with mutant *GRHL2*, although it is unclear why the most prominent skin scaling was found around the margins of the soles. We also noted reduced expression of *TERT* in the affected individuals’ skin and keratinocytes. Overexpression of *GRHL2* in normal keratinocytes increases telomerase activity and increases replicative life span (*TERT* and *PCNA* [MIM 176740]). In contrast, knockdown of *GRHL2* represses the expression of these genes.^12^

The impact of *GRHL2* mutations on cell morphology has been previously described.^4^ Lung epithelial cells transduced with *Grhl2* small hairpin RNA flatten in culture and lose their cuboidal morphology into an expanded cell phenotype. Knocking down *Grhl2* in lung epithelial cell lines leads to downregulation of *Cldn4* and *Cdh1*.^9^ In our subjects’ keratinocytes, immunostaining with E-cadherin showed reduced expression and qPCR showed downregulation of this transcript (Figure S6).

In addition to being expressed in skin, *Grhl2* and *GRHL2* are highly expressed in the inner ear, the lung epithelium, the ureteric bud of the kidney, the olfactory epithelium, the urogenital tract, the gastric mucosa, and human breast cancer cells.^4,8–11,18,27,28^ With regard to the clinical phenotype in our affected individuals, aside from the changes affecting the skin and oral mucosa, the other main features comprised deafness and asthma, although this was variably present. Three subjects (ED-01 IV-4 and IV-5 and ED-02 VI-3) had deafness that developed in early infancy (c.f. the later-onset deafness in other families with GRHL2 haploinsufficiency).^15,16^ Of note, none of the heterozygous carriers of either missense mutation in *GRHL2* had any deafness. The significance of GRHL2 in vertebrate inner-ear development is well established,^16^ but the lack of deafness in the heterozygotes (and some homozygotes) in our pedigrees indicates a different functional effect of the missense mutations. Deafness is not a common feature of ectodermal dysplasia syndromes, although hearing loss can result from abnormalities in p63 and Notch signaling (morphological defects in organ of Corti)^29^ and mutations in connexins 26 and 30 (altered endolymph ion homeostasis).^30^ In contrast, mutagenesis studies in *Grhl2* have indicated a probable different pathophysiology for deafness with enlarged otocysts, absent otoliths, and malformed semicircular canals.^16^

The observation that three of the affected individuals (ED-01 IV-4 and IV-5 and ED-02 IV-2) had clinical symptoms of asthma is also noteworthy because the top enriched GO disease among the downregulated transcripts in our microarray data was asthma (Table S10). Previous in situ hybridization analyses have indicated that *Grhl2* is the only family member that is highly expressed in distal lung epithelium throughout development, although the particular cells expressing *Grhl2* have not been identified, nor has its functional role in the lung epithelium been fully established.^4^ *Grhl1* and *Grhl3*, in contrast, are expressed in the embryonic lung epithelium, but later their expression is reduced in bronchi and bronchioles and is undetectable in the alveolar lung epithelium.^4,27^ The potential relevance of other sequence variants in *GRHL2* to sporadic or familial cases of human asthma and other obstructive-airway diseases remains to be determined.

In summary, GRHL2, a member of a family of highly conserved transcription factors, is implicated in epithelial morphogenesis across a number of species. We have used whole-exome sequencing to identify *GRHL2* mutations underlying an ectodermal dysplasia syndrome in two families, and our data expand the current knowledge about the role of *GRHL2* in human disease and epithelial cell biology.

## Supplemental Data

Document S1. Figures S1–S6 and Tables S1–S13

Document S2. Article plus Supplemental Data

## Web Resources

The URLs for data presented herein are as follows:

Ensembl Genome Browser, http://www.ensembl.org/index.html
Gene Expression Omnibus, http://www.ncbi.nlm.nih.gov/geo/
Gene Ontology Consortium, http://www.geneontology.org/
Online Mendelian Inheritance in Man (OMIM), http://www.omim.org/
Primer3, http://frodo.wi.mit.edu/primer3/
PubMed, http://www.ncbi.nlm.nih.gov/PubMed/
RefSeq, http://www.ncbi.nlm.nih.gov/RefSeq
UCSC Genome Browser, http://genome.ucsc.edu/

## Accession Numbers

The Gene Expression Omnibus accession number for the microarray data reported in this paper is number GSE56486.
