# Supplementary material for: Mutations in GRHL2 Result in an Autosomal-Recessive Ectodermal Dysplasia Syndrome
Source: Am J Hum Genet. 2014 Sep 4;95(3):308–14. doi: 10.1016/j.ajhg.2014.08.001 (PMC4157147; doi:10.1016/j.ajhg.2014.08.001)
Supplement: Document S1. Figures S1–S6 and Tables S1–S13 [file mmc1.pdf]

The American Journal of Human Genetics, Volume *95*

Supplemental Data

Mutations in *GRHL2* Result in an Autosomal-Recessive Ectodermal Dysplasia Syndrome

Gabriela Petrof, Arti Nanda, Jake Howden, Takuya Takeichi, James R. McMillan, Sophia Aristodemou, Linda Ozoemena, Lu Liu, Andrew P. South, Celine Pourreyron, Dimitra Dafou, Laura E. Proudfoot, Hejab Al-Ajmi, Masashi Akiyama, W.H. Irwin McLean, Michael A. Simpson, Maddy Parsons, and John A. McGrath

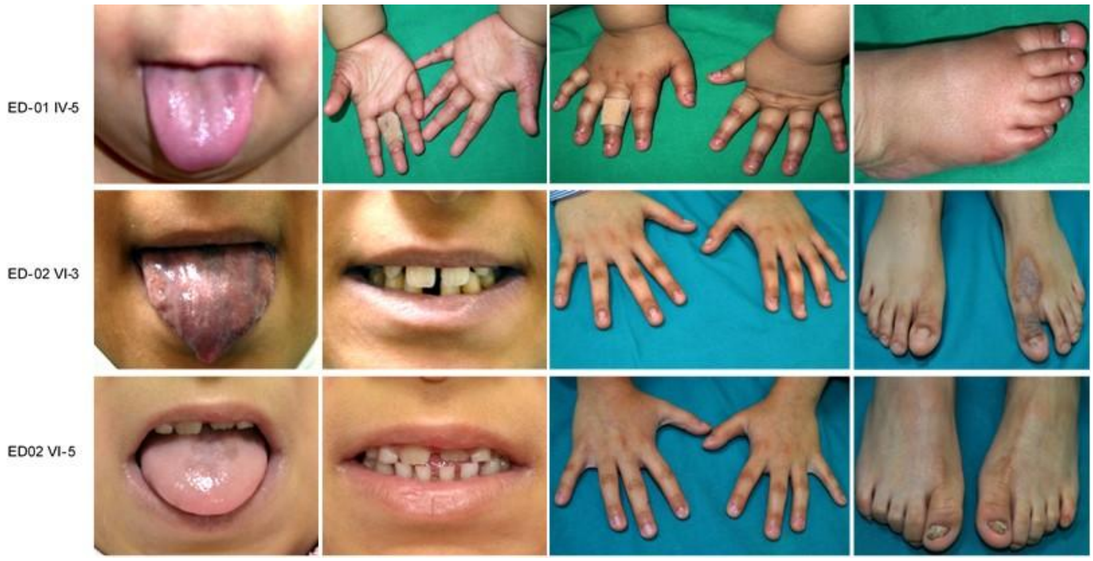

Figure S1. Clinical illustration of this ectodermal dysplasia syndrome. Additional clinical images of other affected members from both pedigrees (See also Figure 1 in main manuscript).

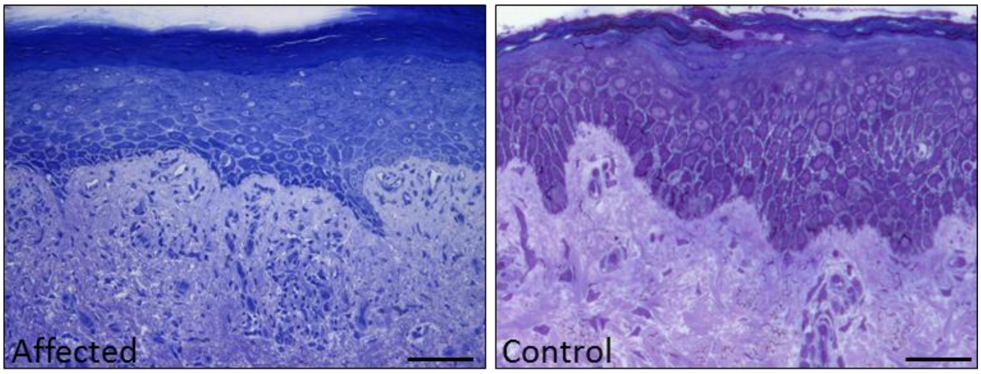

Figure S2. Skin histology. Light microscopy of skin sample from the foot of ED-01 IV-4 shows mild acanthosis and hyperkeratosis (Richardson's stain; scale bar represents 50µm).

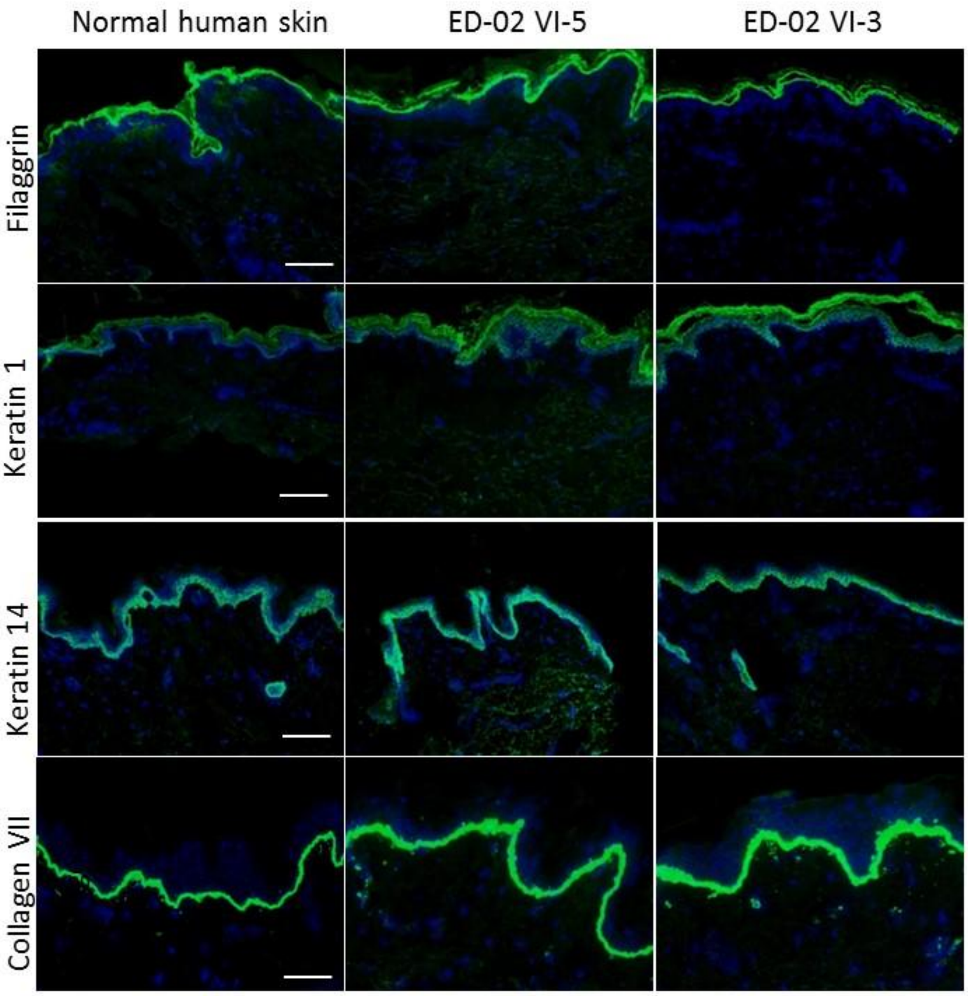

Figure S3. Skin immunolabeling. Immunofluorescence microscopy reveals no major differences in intensity labeling for several epidermal and basement membrane proteins in 2 affected individuals in pedigree ED-02 (right panels) compared to control skin (left panel). Scale bars represent 50 µm. (See Table S13 for antibody details).

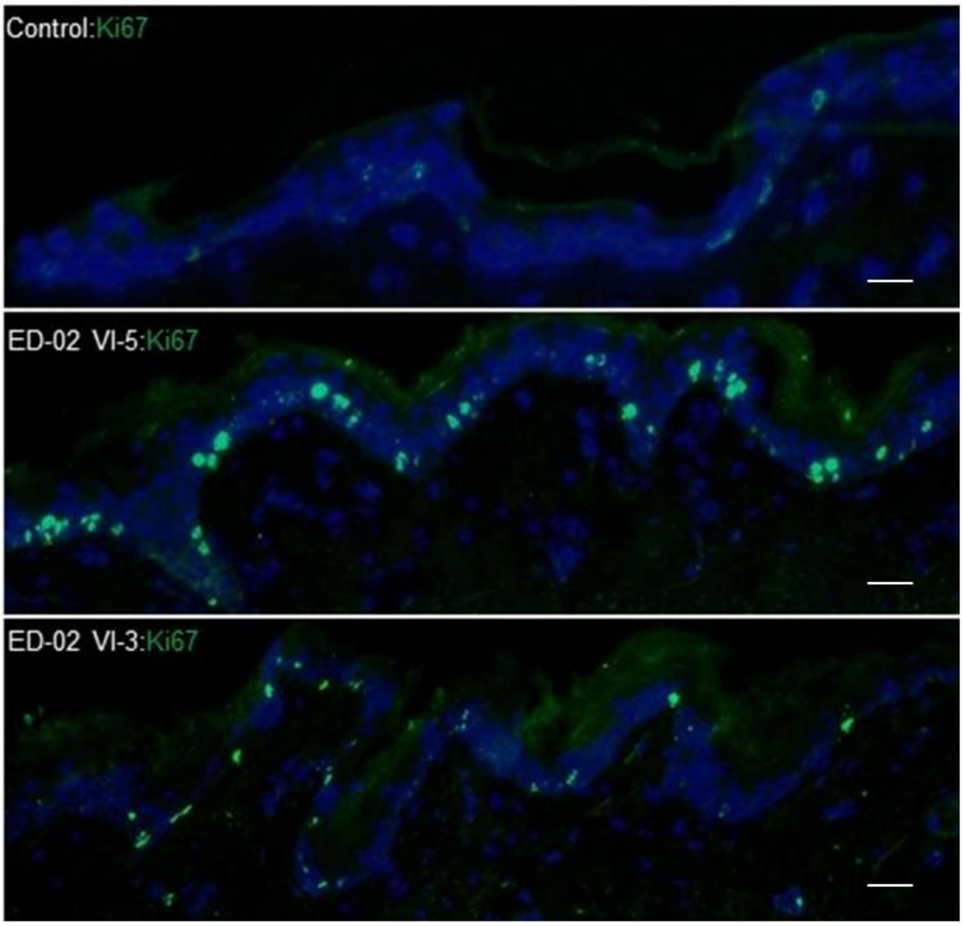

Figure S4. Skin immunolabeling for Ki67. Representative images of control and affected person skin sections stained for the proliferation marker Ki67. Ki-67 labeling is increased in the epidermis of affected individuals. Scale bars represent 50 µm.

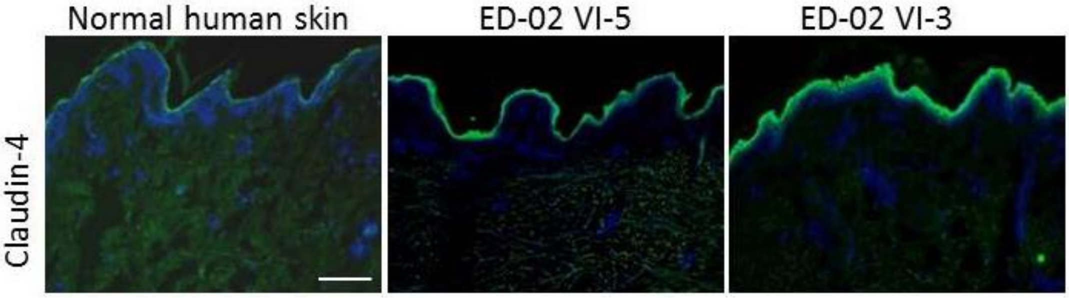

Figure S5. Skin immunolabeling for claudin-4. Immunofluorescence microscopy reveals increased intensity labeling for claudin-4 in two affected individuals in pedigree ED-02 (right panels) compared to control skin (left panel). Scale bars represent 50 µm.

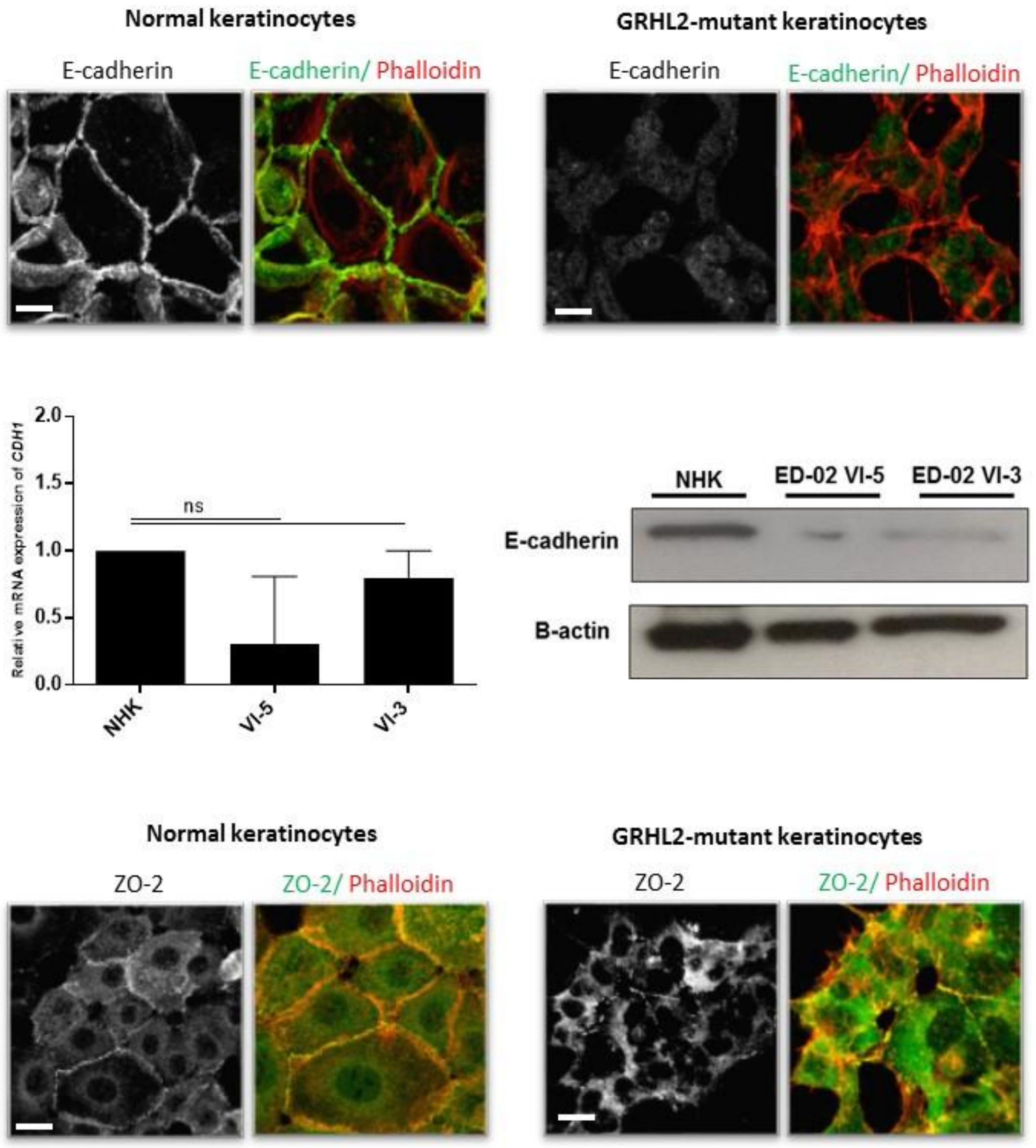

Figure S6. Impact of mutations in *GRHL2* on E-cadherin and zona occludens-2. In cultured keratinocytes staining for the adherens junctional protein E-cadherin appears reduced in the mutant cells with less cell membrane labeling. E-cadherin is reduced at a protein level (Western blotting) although gene expression is not significantly reduced by qPCR. The membranous localization of the tight junction protein zona occludens-2 (ZO-2) is also diminished in mutant keratinocytes. Phalloidin labels the actin cytoskeleton. Scale bar represents 20 µm.

TABLE S1: EXOME SEQUENCING DETAILS

**(Patient ED-01 IV-4)**	
Total sequence reads	18908110
Quality control passed aligned reads	14667221
%	77.57
Quality control passed aligned reads +/- 150bp	17112569
%	90.50
Mean read depth	26.80
% of exome > 1x reads	96.82
% of exome > 5x reads	89.51
% of exome >10x reads	76.05
% of exome >20x reads	49.10
**(Patient ED-02 VI-3)**	
Total sequence reads	79571546
Quality control passed aligned reads	56980960
%	71.61
Quality control passed aligned reads +/- 150bp	64235288
%	80.73
Mean read depth	105.46
% of exome > 1x reads	98.12
% of exome > 5x reads	96.44
% of exome >10x reads	94.64
% of exome >20x reads	89.92

TABLE S2. FILTERED EXOME VARIANTS

Patient ED-01 IV:4					
**Gene**	**Gene name**	**Mutation**	**Size of homozygous block (Mb)**	**SIFT**	**Poly-phen2**
CLCNKA	Chloride channel protein ClC-Ka isoform 2	Splicing	0.834	-	-
GDAP1	Ganglioside-induced differentiation-associated	Nonsynonymous SNV	56.9	Damaging (0.03)	Benign (0.139)
GRHL2	Grainyhead-like protein 2 homolog	Nonsynonymous SNV	56.9	Tolerated (0.52)	Probably damaging (0.994)
RRM2B	Gibonucleoside-diphosphate reductase subunit M2	Nonsynonymous SNV	56.9	Tolerated (0.07)	Benign (0)
CAND1	Cullin-associated and neddylation	Splicing	12.5	-	-
MDM1	Nuclear protein MDM1	Splicing	12.5	-	-
NIN	Ninein isoform 1	Nonsynonymous SNV	14.8	Damaging (0.05)	Probably damaging (0.961)
GOLGA6L6	Golgin A6 family-like 6	Nonsynonymous SNV	0.000282	Tolerated (0.08)	Probably damaging (0.998)
APOL3	Apolipoprotein L, 3	Nonsynonymous SNV	19.5	Tolerated (0.07)	Probably damaging (0.993)
MKL1	MKL (megakaryoblastic leukemia)/myocardin-like	Nonsynonymous SNV	19.5	Damaging (0.05)	Probably damaging (1)
**Patient ED-02 VI:3**					
**Gene**	**Gene name**	**Mutation**	**Size of homozygous block (Mb)**	**SIFT**	**Poly-phen2**
PROM1	Prominin 1	Nonsynonymous SNV	1.88	Tolerated (0.54)	Possibly damaging (0.513)

TBC1D1	TBC1 (tre-2/USP6, BUB2, cdc16) domain family, member 1	Splicing	0.0464	-	-
RAB11FIP1	RAB11 family interacting protein 1 (class I)	Nonsynonymous SNV	21.8	Tolerated (0.36)	Benign (0.001)
GGH	Gamma-glutamyl hydrolase (conjugase, folylpolygammaglutamyl hydrolase)	Nonsynonymous SNV	36.6	Tolerated (0.11)	Probably damaging (0.998)
GRHL2	Grainyhead-like protein 2 homolog	Nonsynonymous SNV	19.6	Tolerated (0.28)	Probably damaging (0.984)
DUSP14	Dual specificity phosphatase 14	splicing	0.153	-	-

Legend: Hom: homozygous, SNV: single nucleotide variation.

This table shows the size of the homozygous blocks of homozygosity surrounding each novel homozygous mutation and the predicted pathogenicity of the missense mutations. The sizes of the putative regions of homozygosity were estimated by the distance between the delimiting heterozygous variants identified by whole-exome sequencing. The SIFT score ranges from 0 to 1. Values above 0.05 are predicted to be tolerated and values below 0.05 are considered damaging. The Polyphen-2 scoring system differs with values greater than 0.5 regarded as possibly damaging and below 0.5 reported as benign. SNV: single nucleotide variation.

Table S3. Enriched GO Pathways amongst up-regulated transcripts.

#	Pathways	p-Value
**1**	Protein folding and maturation_POMC processing	7.53e-22
**2**	SCAP/SREBP transcriptional control of cholesterol and FA biosynthesis	1.653-09
**3**	Regulation of metabolism_Bile acid regulation of glucose and lipid metabolism via FXR	4.40e-06
**4**	Role of diethylhexyl Phthalate and Tributylin in fat cell diffentiation	8.14e-06
**5**	Cytoskeleton remodeling_Keratin filaments	3.71e-05
**6**	Cell cycle_Spindle assembly and chromosome separation	2.01e-04
**7**	Phenylalanine metabolism/Rodent version	3.37e-04
**8**	Phenylalanine metabolism	3.74e-04
**9**	Pyruvate metabolism	4.37e-04
**10**	Cell adhesion_tight junctions	7.40e-04

N.B. Protein folding and maturation_POMC processing was enriched in several other unrelated experiments on the microarray and may be a spurious finding.

Table S4. Enriched GO Pathways amongst down-regulated transcripts.

#	Pathways	p-Value
**1**	Immune response_HSP60 and HSP70/TLR signaling pathway	7.57e-08
**2**	Immune response_MIF-induced cell adhesion, migration and angiogenesis	1.30e-07
**3**	Reproduction_GnRH signaling	7.49e-07
**4**	Immune response IL-5 signaling	1.15e-06
**5**	Stellate cells activation and liver fibrosis	1.39e-06
**6**	Immune response_IL-18 signaling	1.40e-06
**7**	Immune response_Immunological synapse formation	1.81e-06
**8**	Immune response_C5a signaling	3.34e-06
**9**	Development_GM-CSF signaling	3.58e-06
**10**	Immune response_Oncostatin M signaling via MAPK in human cells	4.36e-06

Table S5. Enriched GO Processes amongst up-regulated transcripts.

#	Processes	p-Value
**1**	Lipid metabolic process	7.01e-23
**2**	Small molecule metabolic process	2.97e-17
**3**	Cellular lipid metabolic process	4.53e-12
**4**	Fatty acid metabolic process	1.52e-11
**5**	Metabolic process	9.03e-10
**6**	Ferric iron transport	5.51e-09
**7**	Fatty acid biosynthetic process	7.41e-09
**8**	Triglyceride biosynthetic process	9.95e-08
**9**	Cellular response to cAMP	7.95e-07
**10**	Response to lithium ion	8.22e-07

Table S6. Enriched GO Processes amongst down-regulated transcripts.

#	Processes	p-Value
**1**	Cell adhesion	3.77e-17
**2**	Extracellular matrix organization	4.81e-17
**3**	Inflammatory response	4.54e-16
**4**	Innate immune response	2.52e-15
**5**	Immune response	1.25e-14
**6**	Response to estradiol stimulus	2.78e-14
**7**	Response to lipopolysaccharide	7.46e-14
**8**	Neutrophil chemotaxis	3.31e-13
**9**	Interferon-gamma-mediated signaling pathway	4.79e-13
**10**	Response to mechanical stimulus	7.05e-12

Table S7. Enriched GO Networks amongst up-regulated transcripts.

#	Networks	p-Value
**1**	Signal transduction_Neuropeptide signaling pathways	1.54e-08
**2**	Cytoskeleton_Intermediate filaments	2.05e-05
**3**	Reproduction_Progesterone signaling	4.79e-05
**4**	Cell adhesion_Cell junctions	8.09e-05
**5**	Signal transduction_WNT signaling	2.18e-04
**6**	Reproduction_Spermatogenesis, motility and copulation	4.09e-04
**7**	Cytoskeleton_Actin filaments	2.05e-03
**8**	Regulation of metabolism_Bile acid regulation of lipid metabolism and negative FXR-dependent regulation of bile acids concentration	3.10e-03
**9**	Transport_Iron transport	4.49e-03
**10**	Proteolysis_Connective tissue degradation	8.42e-03

Table S8. Enriched GO Networks amongst down-regulated transcripts.

#	Networks	p-Value
**1**	Cell adhesion_Cell matrix interactions	6.79e-13
**2**	Cell adhesion_Platelet-endothelium-leucocyte interactions	3.71e-11
**3**	Immune response_Antigen presentation	2.84e-06
**4**	Immune response_Th17-derived cytokines	5.10e-06
**5**	Inflammation_IL-4 signaling	1.56e-05
**6**	Development_Regulation of angiogenesis	6.89e-05
**7**	Development_EMT_Regulation of epithelial-to-mesenchymal transition	8.99e-05
**8**	Cell adhesion_Integrin-mediated cell-matrix adhesion	1.05e-04
**9**	Immune response_IL-5 signaling	1.32e-04
**10**	Reproduction_Gonadotropin regulation	1.43e-04

Table S9. Enriched GO Diseases amongst up-regulated transcripts.

#	Diseases	p-Value
**1**	Adrenal insufficiency	2.01e-26
**2**	Cushing syndrome	9.46e-21
**3**	Pain	4.53e-19
**4**	Eclampsia	6.74e-19
**5**	Hepatitis, autoimmune	2.28e-18
**6**	Fatigue Syndrome, Chronic	3.07e-17
**7**	Osteoporosis	1.08e-17
**8**	Mesothelioma	1.49e-17
**9**	Obesity, Morbid	3.16e-17
**10**	Depressive disorder	1.31e-16

Table S10. Enriched GO Diseases amongst down-regulated transcripts.

#	Diseases	p-Value
1	Asthma	2.28e-28
2	Arthritis, Rheumatoid	1.60e-26
3	Wounds and Injuries	1.04e-25
4	Inflammation	1.06e-22
5	Fibrosis	2.32e-21
6	Arthritis	1.54e-20
7	Pulmonary disease, chronic obstructive	2.06e-19
8	Colitis, ulcerative	2.12e-18
9	Melanoma, cutaneous malignant	3.47e-18
10	Carcinoma, non-small cell lung	4.36e-18

Table S11. Skin differentiation/barrier gene expression levels. Gene expression levels were compared using RNA from whole skin of 3 individuals from the two pedigrees, as well as immortalized keratinocytes and primary fibroblasts from one affected subject (ED-02 VI-5) relative to control samples. ND = Not Detected.

Gene symbol	Transcriptomic data (fold change)		Qualitative Real-time PCR				
	ED-02 VI-5 whole skin	ED-02 VI-3 Whole skin	ED-02 VI-5 Immortalized keratinocytes	ED-02 VI-5 Primary fibroblasts	ED-01 IV-4 Whole skin	ED-02 VI-5 Whole skin	ED-02 VI-3 Whole skin
GRHL1	1.2	1.4	1.64	ND	1.17	8.3	2.76
GRHL2	0.78	0.66	0.2	ND	0.37	0.76	0.37
GRHL3	1.16	1.39	1.52	ND	1.87	2.73	4.06
CDH1	1.17	1.1	0.5	ND	0.44	1.19	3.2
KRT10	0.89	0.91	0.25	ND	0.99	0.79	0.89
KRT14	1.21	1.15	3.2	ND	1.23	2.43	2.85
S100A8	0.38	0.22	0.50	ND	0.59	0.12	0.48
IVL	1.65	1.42	0.24	ND	0.70	13.82	3.73
FLG	1.05	1.13	0.28	ND	0.05	11.65	4.86
CLDN3	1.47	2.38	4.70	ND	19.27	11.71	8.33
CLDN4	1.8	1.94	1.87	ND	1.61	16.07	3.45
RAB25	1.38	1.47	2.79	0.14	1.14	1.81	2.85
TSLP	0.43	1.82	0.61	0.34	0.62	0.75	1.15
DSG1	1.1	1.13	9.41	ND	0.71	1.42	3.58
TJP2	1.32	1.1	ND	1.0	1.13	1.05	1.31
TERT	0.84	0.68	0.66	ND	ND	0.21	0.12
AQP7	52.7	119.5	8.24	ND	5.35	81.6	105.36
TGM1	1.0	1.38	0.03	ND	0.01	0.95	1.45

These data allow for verification of transcriptomic data by qPCR, confirmation of gene expression changes for different mutations in *GRHL2*, and insight into gene expression similarities and differences in pure cell populations (keratinocytes/fibroblasts) and well as in *in vivo* differentiated skin. The homozygous missense mutations in *GRHL2* are associated with upregulation of *GRHL1*, *GRHL3*, *KRT14* (keratin 14), *CLDN3* (claudin 3), *CLDN4* (claudin 4), *RAB25* (Ras-related protein Rab-25) and *AQP7* (aquaporin 7). The mutations also are found in association with reduced expression of *GRHL2*, *KRT10* (keratin 10), and *TERT* (telomerase reverse transcriptase). Inter-individual differences were seen for *CDH1* (E-cadherin), *IVL* (involucrin), *FLG* (profilaggrin), *TSLP* (thymic stromal lymphopoietin), *DSG1* (desmoglein1) and *TGM1* (transglutaminase 1).

Table S12. List of Taqman Gene expression assays used for qPCR. Following the extraction and quantification procedure, cDNA was produced by reverse transcription (RT) of RNA using the High Capacity Reverse Transcription kit (Applied Biosystems) according to the manufacturer's instructions. TaqMan_ Gene Expression Assays were purchased from Applied Biosystems (Warrington, U.K.) and used as directed. Samples were run and analyzed using the 7000HT Real-Time PCR System (Applied Biosystems). Expression values for each primer set were normalized to 18S ribosomal RNA (rRNA) gene values. The relative expression in the affected people samples vs. respective control samples was then determined using the DDCt method.

Gene symbol	Taqman Gene expression Assay
***GRHL1* [MIM 609786]**	Hs01119372_m1
***GRHL2* [MIM 609576]**	Hs00227745_m1
***GRHL3* [MIM 609317]**	Hs00297962_m1

***FLG* [MIM 135490]**	Hs00856927_g1
***IVL* [MIM 147360]**	Hs00846307_s1
***CDH1* [MIM 192090]**	Hs01023894_m1
***KRT10* [MIM 148080]**	Hs00166289_m1
***KRT14* [MIM 148066]**	Hs00265033_m1
***S100A8* [MIM 123885]**	Hs00374264_g1
***CLDN3* [MIM 602910]**	Hs00265816_s1
***CLDN4* [MIM 602909]**	Hs00976831_s1
***RAB25* [MIM 612942]**	Hs01040784_m1
***TSLP* [MIM 607003]**	Hs00263639_m1
***DSG1* [MIM 125670]**	Hs00355084_m1
***TJP2* [MIM 607709]**	Hs00910543_m1
***TERT* [MIM 187270]**	Hs00972656_m1
***AQP7* [MIM 602974]**	Hs00357359_m1
***TGM1* [MIM 190195]**	Hs01070310_m1

Table S13: Antibodies used in this study

rabbit anti-GRHL2	1:500	(HPA004820, Sigma-Aldrich, UK)
mouse anti-claudin-4	1:200	(3E2C1, Life Technologies, Paisley, UK)
mouse anti-collagen VII	1:150	(C6805, Sigma-Aldrich)
mouse anti-E-cadherin	1:1,000	(HECD1, Life Technologies)
mouse anti-keratin 14	1:100	(LL02, Bio-Rad AbD Serotec, San Francisco, CA)
rabbit anti-keratin 5	1:100	(ab24647, Abcam, Cambridge, UK)
mouse anti-cytokeratin 1	1:20	(34Bb4, Enzo Diagnostics, New York, NY)
mouse anti-keratin 10	1:20	(LHP1, Abnova)
mouse anti-filaggrin	1:20	(FLG01, Abcam)
mouse anti-involucrin	1:20	(SY5, Santa Cruz Biotechnology, Santa Cruz, CA)
rabbit anti-ki67	1:500	(ab15580, Abcam)
mouse β-actin	1:10,000	(AC-74, Abcam)
phalloidin-Alexa-568	1:400	(Life Technologies).
