# Supplementary material for: Mutations in GRHL2 Result in an Autosomal-Recessive Ectodermal Dysplasia Syndrome
Source: Am J Hum Genet. 2014 Sep 4;95(3):308–14. doi: 10.1016/j.ajhg.2014.08.001 (PMC4157147; doi:10.1016/j.ajhg.2014.08.001)
Supplement: Document S2. Article plus Supplemental Data [file mmc2.pdf]

Mutations in *GRHL2* Result in an Autosomal-Recessive Ectodermal Dysplasia Syndrome

Gabriela Petrof,[1] Arti Nanda,[2] Jake Howden,[3] Takuya Takeichi,[1,4] James R. McMillan,[5] Sophia Aristodemou,[5] Linda Ozoemena,[5] Lu Liu,[5] Andrew P. South,[6] Celine Pourreyron,[6] Dimitra Dafou,[7] Laura E. Proudfoot,[1] Hejab Al-Ajmi,[2] Masashi Akiyama,[4] W.H. Irwin McLean,[6] Michael A. Simpson,[8] Maddy Parsons,[3] and John A. McGrath[1,6,*]

Grainyhead-like 2, encoded by *GRHL2*, is a member of a highly conserved family of transcription factors that play essential roles during epithelial development. Haploinsufficiency for *GRHL2* has been implicated in autosomal-dominant deafness, but mutations have not yet been associated with any skin pathology. We investigated two unrelated Kuwaiti families in which a total of six individuals have had lifelong ectodermal defects. The clinical features comprised nail dystrophy or nail loss, marginal palmoplantar keratoderma, hypodontia, enamel hypoplasia, oral hyperpigmentation, and dysphagia. In addition, three individuals had sensorineural deafness, and three had bronchial asthma. Taken together, the features were consistent with an unusual autosomal-recessive ectodermal dysplasia syndrome. Because of consanguinity in both families, we used whole-exome sequencing to search for novel homozygous DNA variants and found *GRHL2* mutations common to both families: affected subjects in one family were homozygous for c.1192T>C (p.Tyr398His) in exon 9, and subjects in the other family were homozygous for c.1445T>A (p.Ile482Lys) in exon 11. Immortalized keratinocytes (p.Ile482Lys) showed altered cell morphology, impaired tight junctions, adhesion defects, and cytoplasmic translocation of GRHL2. Whole-skin transcriptomic analysis (p.Ile482Lys) disclosed changes in genes implicated in networks of cell-cell and cell-matrix adhesion. Our clinical findings of an autosomal-recessive ectodermal dysplasia syndrome provide insight into the role of GRHL2 in skin development, homeostasis, and human disease.

Grainyhead-like 2 (GRHL2) is a mammalian homolog of *Drosophila* protein grainy head (GRH), which, along with GRHL1 and GRHL3, has a role in epithelial morphogenesis.[1,2] This family of transcription factors controls the development and differentiation of multicellular epithelia by regulating genes germane to cell junction formation and proliferation.[3,4] Biologically, GRHL2 contributes to formation of the epithelial barrier and wound healing, as well as neural-tube closure, maintenance of the mucociliary airway epithelium, and tumor suppression.[5–11] *GRHL2* (MIM 608576) has been shown to regulate *TERT* (MIM 187270) expression and to enhance proliferation of epidermal keratinocytes; it also impairs keratinocyte differentiation through transcription inhibition of genes clustered at the epidermal differentiation complex[12] and regulates epithelial morphogenesis by establishing functional tight junctions.[13]

GRHL2 is also present in the cochlear duct,[14] and mutations in human *GRHL2* have been found in progressive autosomal-dominant hearing loss (DFNA28 [MIM 608641]),[15,16] and other polymorphic sequence variants in *GRHL2* have been implicated in age-related hearing impairment and noise-induced hearing loss.[17–19] To date, however, the role of GRHL2 in skin biology has not been well established. Causing severe facial and neural-tube defects, *Grhl2* knockout is embryonically lethal in mice,[17,20] and mutant zebrafish display inner-ear defects and abnormal swimming positions.[18] In contrast, *Grhl1*$^{-/-}$ mice show hair loss and palmoplantar keratoderma, as well as abnormal desmosome cell junctions and dysregulated terminal differentiation in keratinocytes.[21] Moreover, *Grhl3*$^{-/-}$ embryos fail to establish a normal epidermal barrier and display defective embryonic wound repair.[22] Thus, unlike for GRHL1 and GRHL3, there is currently a lack of data associating GRHL2 with skin pathology. In this report, however, we have identified two families in which affected subjects have developmental defects affecting skin, oral mucosa, and teeth (as well as hearing and lungs), thus implicating *GRHL2* in an autosomal-recessive ectodermal dysplasia syndrome.

We investigated two unrelated Kuwaiti families, both consanguineous, in which clinically similar features were present in a total of six affected individuals (Figures 1A and 1B). The clinical features were noted in early infancy and comprised short stature ($\leq 25^{th}$ percentile), nail dystrophy and/or loss, oral mucosa and/or tongue pigmentation, abnormal dentition (delay, hypodontia, enamel hypoplasia), keratoderma affecting the margins of the palms

[1]St. John's Institute of Dermatology, King's College London, Guy's Campus, London SE1 9RT, UK; [2]As'ad Al-Hamad Dermatology Center, Al-Sabah Hospital, Kuwait City 13001, Kuwait; [3]Randall Division of Cell and Molecular Biophysics, King's College London, Guy's Campus, London SE1 9RT, UK; [4]Department of Dermatology, Nagoya University Graduate School of Medicine, Nagoya 466-8560, Japan; [5]National Diagnostic Epidermolysis Bullosa Laboratory, Viapath, St. Thomas' Hospital, London SE1 7EH, UK; [6]Dermatology and Genetic Medicine, College of Life Sciences and College of Medicine, Dentistry, and Nursing, University of Dundee, Dundee DD1 5EH, UK; [7]Department of Genetics, Development, and Molecular Biology, School of Biology, Aristotle University, Thessaloniki 54124, Greece; [8]Department of Medical and Molecular Genetics, King's College London School of Medicine and Guy's Hospital, London SE1 9RT, UK
*Correspondence: john.mcgrath@kcl.ac.uk
http://dx.doi.org/10.1016/j.ajhg.2014.08.001. ©2014 The Authors
This is an open access article under the CC BY license (http://creativecommons.org/licenses/by/3.0/).

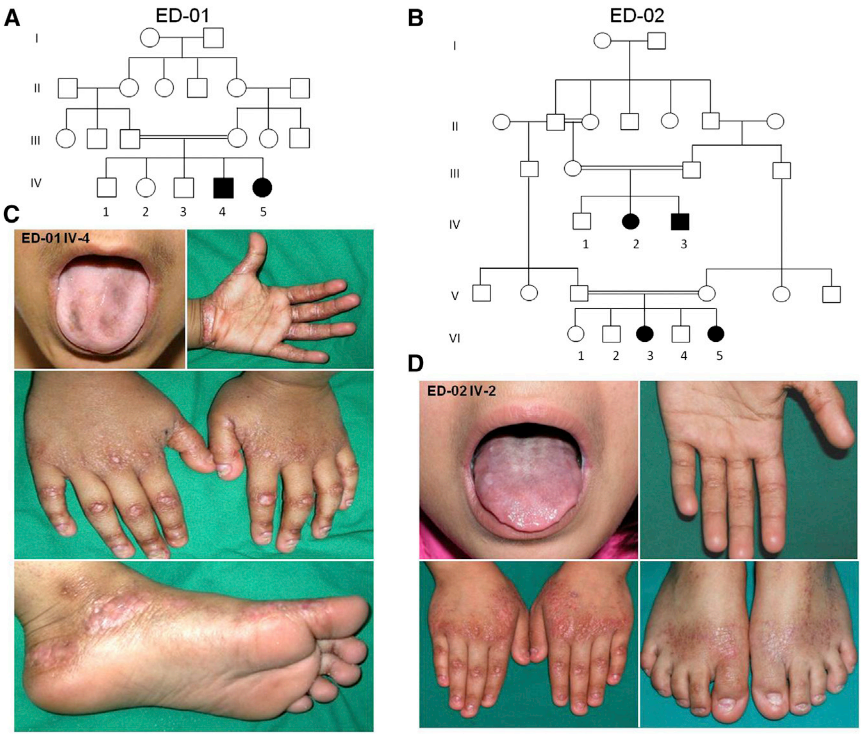

Figure 1. Pedigrees and Clinical Features of This Autosomal-Recessive Ectodermal Dysplasia Syndrome

(A and B) Two unrelated consanguineous pedigrees with a total of six affected individuals.

(C and D) An affected 8-year-old male (from pedigree ED-01) and an affected 12-year-old female (from pedigree ED-02) both show features of tongue hyperpigmentation, skin thickening around the margins of the palms and soles, hypoplastic finger and toe nails, knuckle pads on the fingers, and atrophic wrinkling on the dorsal aspects of the hands and feet. Additional clinical images from other subjects are shown in Figure S1.

and soles, and focal hyperkeratosis of the dorsal aspects of the hands and feet (Figures 1C and 1D; Figure S1, available online). No individual showed any wound-healing defect, blistering tendency, hair or sweating abnormalities, or other developmental anomalies. Two affected sisters (ED-02 VI-3 and VI-5) had dysphagia with evident esophageal strictures. Three individuals (ED-01 IV-4 and IV-5 and ED-02 VI-3) developed sensorineural deafness in early infancy, and three others (ED-01 IV-4 and IV-5 and ED-02 IV-2) had bronchial asthma. One individual (ED-01 IV-4) had severe iron-deficiency anemia requiring blood transfusion. Laboratory tests (full blood count, serum biochemistry, immunoglobulin levels, and thyroid-function tests) were otherwise within the normal range for all affected individuals. None of the parents had any skin, hair, teeth, nail, or hearing abnormalities.

To investigate the etiology of the condition, we first assessed lesional skin biopsies taken from three affected individuals (ED-01 IV-4 and ED-02 VI-3 and VI-5) by using immunohistochemistry and transmission electron microscopy. The subjects' legal guardians provided written informed consent according to a protocol approved by the St. Thomas' Hospital Ethics Committee (Molecular basis of inherited skin disease: 07/H0802/104). Blood and skin samples (ellipse of skin taken under local anesthesia by 1% lignocaine) were obtained in adherence to the Declaration of Helsinki guidelines. Light microscopy showed mild acanthosis and hyperkeratosis (Figure S2), but transmission electron microscopy of the skin was unremarkable—it showed no clear abnormalities in keratinocytes, hemidesmosomes, or desmosome cell-cell junctions. Likewise, skin immunolabeling using a panel of antibodies that target basement membrane extracellular matrix proteins (collagen IV and collagen VII), epidermal-adhesion-associated proteins (desmoglein 1, keratin 1, keratin 14, and desmoplakin), and markers of terminal differentiation (filaggrin) showed normal intensity and labeling patterns when affected skin from two affected individuals was compared to skin from a normal control subject (Figure S3; see Supplemental Data for methods and antibody details). However, we noted increased staining for the proliferation marker Ki-67 in the epidermis (Figure S4). Collectively, the skin-biopsy findings were not diagnostic for any known inherited skin disease.

We then used whole-exome sequencing to identify a candidate gene or genes. We extracted genomic DNA from peripheral blood from two affected individuals (ED-01 IV-4 and ED-02 VI-3). We performed whole-exome capture by using in-solution hybridization (Agilent All Exon Kit V4) and generated sequencing on the Illumina HiSeq 2000. Resulting reads were aligned to the reference human genome (UCSC Genome Browser hg19, GRCh37) with the Novoalign software package (Novocraft Technologies). Duplicate reads, resulting from PCR clonality or optical duplicates, and reads mapping to multiple locations were excluded from downstream analysis. A summary of exome-coverage data is presented in Table S1. Sixteen previously unreported homozygous mutations were identified (ten in family ED-01 and six in family ED-02). The only gene containing homozygous variants common to both subjects was *GRHL2* (Table S2). The respective mutations were c.1192T>C (p.Tyr398His) in exon 9 and c.1445T>A (p.Ile482Lys) (RefSeq NM_024915.3) in exon 11. These mutations were confirmed by Sanger sequencing (Figures 2A and 2B) and were also shown to segregate with the disease phenotype in other affected pedigree members. Both mutations are located in the DNA binding site of *GRHL2* (Figure 2C) and are predicted to be "probably damaging" by PolyPhen-2 analysis (scores 0.984 and 0.994 for

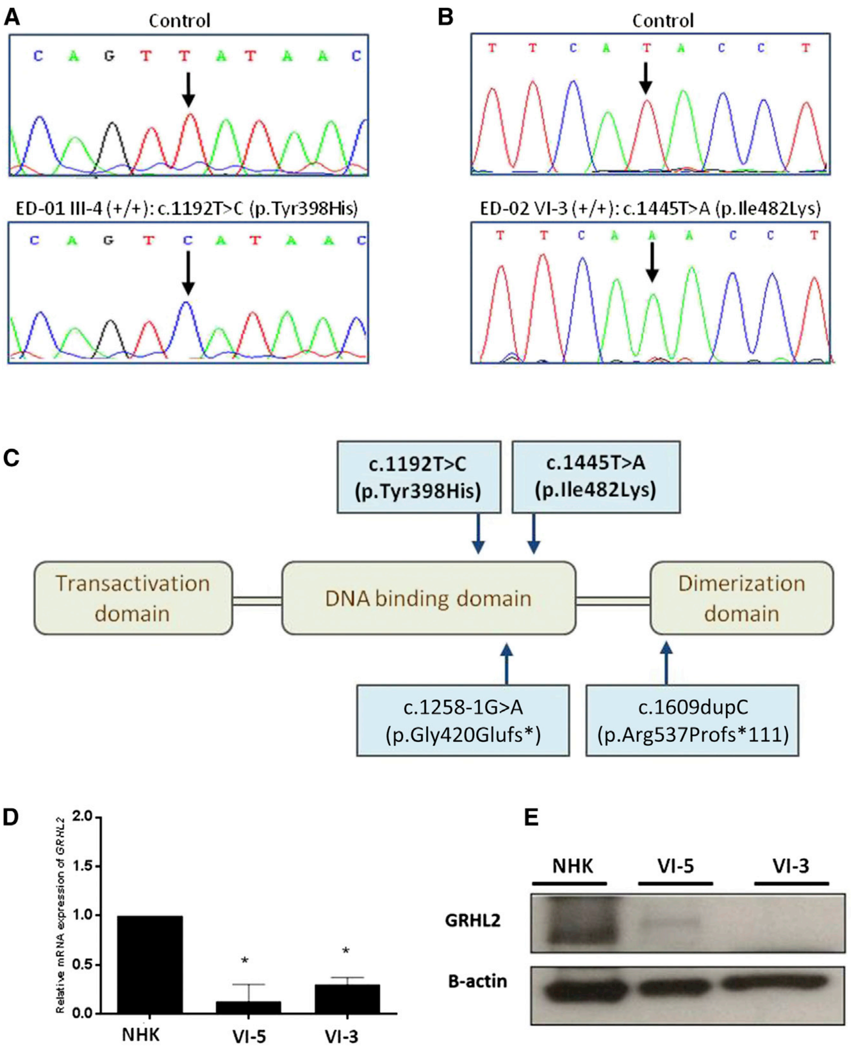

Figure 2. Autosomal-Recessive Mutations in *GRHL2* Lead to Reduced Gene Expression and Protein Levels

(A and B) Sanger sequencing confirmed the presence of different homozygous missense mutations in *GRHL2* in affected subjects from both pedigrees.

(C) Schematic representation of the functional domains of GRHL2. The recessive missense mutations we identified (top) are located within the DNA binding domain; the previously reported heterozygous splice-site or deletion mutations that cause autosomal-dominant deafness are also illustrated (bottom).

(D) qPCR for *GRHL2* expression in cultured keratinocytes showed reduced expression in two affected subjects from pedigree ED-02 (*$p < 0.05$ in comparison to control cells). Error bars represent the SD from three independent experiments.

(E) Immunoblotting using cultured keratinocyte whole-cell lysates revealed markedly reduced or undetectable amounts of GRHL2 in these same individuals.

c.1192T>C and c.1445T>A, respectively). Neither variant has been observed by the 1000 Genomes Project or detected in ~1,200 control in-house exomes or in 260 ethnically matched control chromosomes.

GRHL2 can be detected in the nuclear fraction of normal human keratinocytes in culture (but is subsequently lost in cell senescence).[23] We cultured keratinocytes from two of the skin biopsies (ED-02 VI-3 and ED-02 VI-5) by standard methods and used these cells to examine *GRHL2* expression and GRHL2 localization (see Supplemental Data for methods); both were found to be reduced (Figures 2D and 2E). We then isolated primary keratinocytes from one of the affected individuals (ED-02 VI-5) and immortalized these cells at passage 1 (see Supplemental Data for methods). The phenotype of these cells was assessed by confocal microscopy. *GRHL2* mutant cells showed a less cuboidal, elongated phenotype and failed to form intact cell junctions, as seen in control immortalized keratinocytes. Notably, there was a reduction in cell membrane labeling for E-cadherin (adherens junctions) and zona-occludens-2 (tight junctions) (Figure S5). GRHL2 staining in control keratinocytes was seen both at cell-cell contact areas and within the nucleus (Figure 3A), whereas in mutant cells, the signal was not at the periphery and instead showed a fragmented punctate nuclear localization (Figure 3B). To assess the effects of the mutations on keratinocyte cell function, we also performed assays of cell adhesion and de-adhesion (see Supplemental Data for methods). No differences were noted for cell adhesion between mutant and control cells (Figure 3C), but mutant cells detached from fibronectin much faster than normal human keratinocyte controls after exposure to trypsin (Figure 3D).

Next, we assessed the transcriptome profile by using RNA extracted from whole skin from two individuals in pedigree ED-02. RNA from healthy control skin was obtained from discarded abdominoplasty tissue from plastic surgeons and used as four pooled samples. RNA extraction was performed with the Ambion mirVana miRNA Isolation kit (Invitrogen) according to the manufacturer's instructions. RNA was amplified with the Illumina TotalPrep RNA Amplification Kit, and subsequent gene-expression profiling was performed with the Illumina array HumanHT-12 v4 Expression BeadChip Kit according to the manufacturer's instructions. Gene-expression data were then analyzed with GenomeStudio software (Illumina). A prefiltering set was determined for significantly modulated expression (detection p value < 0.01; signal intensity fold change ≥ 2.0) between affected and control skin. A comprehensive functional-enrichment analysis was then performed with (1) the Database for Annotation, Visualization, and Integrated Discovery (v.6.7), based on the Gene

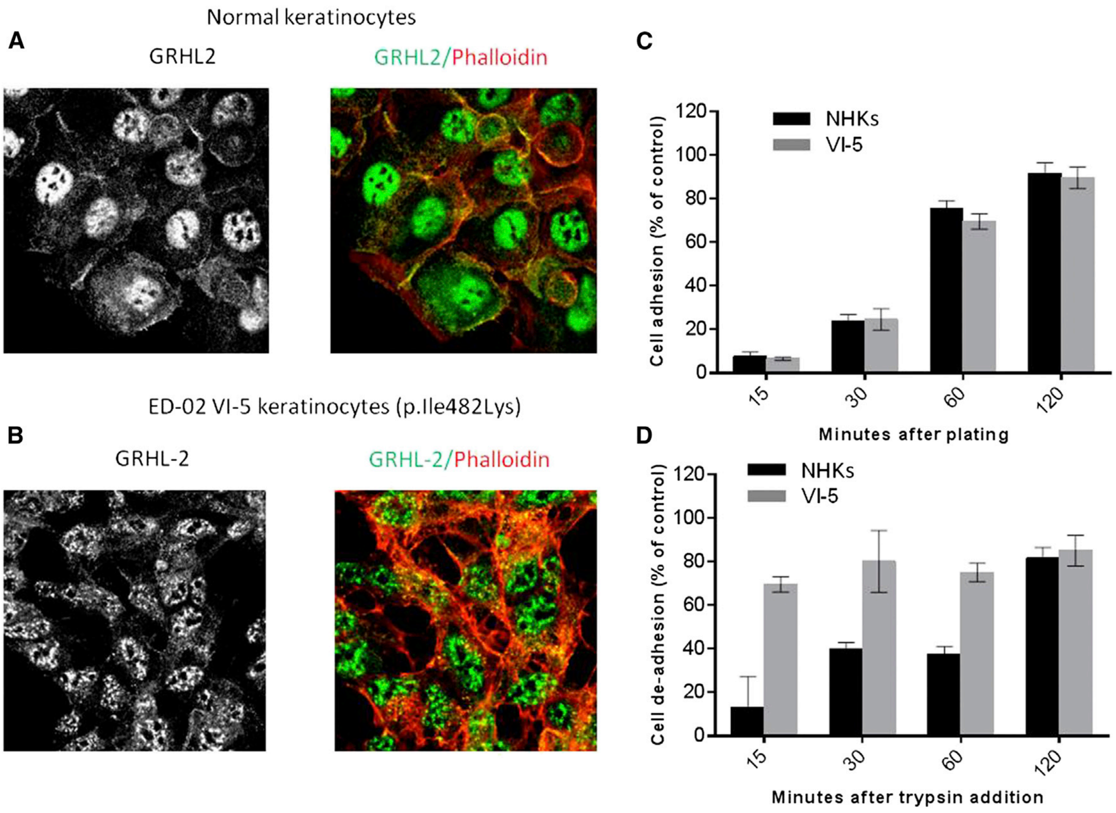

Figure 3. Impact of *GRHL2* Mutations on Keratinocyte Cell Biology

(A) Confocal microscopy in normal keratinocytes revealed nuclear, cytoplasmic, and membranous labeling for an antibody raised against GRHL2.

(B) In contrast, keratinocytes from an affected subject showed an altered pattern of antibody localization within the nucleus and a lack of any cell membrane labeling.

(C) Cell-adhesion assays showed no difference between wild-type and mutant keratinocytes. Error bars represent the SD from three independent experiments.

(D) In contrast, mutant cells showed more rapid detachment in trypsin de-adhesion assays. Error bars represent the SD from three independent experiments. NHK stands for normal human keratinocyte.

Ontology (GO) database (see Web Resources), and (2) the GeneGo Metacore software (Thomson Reuters), a systems-biology analysis tool based on a curated database of human protein-protein and protein-DNA interactions, transcription factors, signaling, and metabolic pathways. Comparison of the affected individuals' skin with the skin of healthy age- and site-matched control individuals identified 1,457 gene transcripts that were significantly altered: 668 upregulated ($\geq$2-fold change) and 789 downregulated ($\leq$0.5-fold change) transcripts for ED-02 VI-5. For ED-02 VI-3, 1,141 gene transcripts were altered: 466 upregulated and 675 downregulated. Of these changes in gene expression, 359 upregulated and 344 downregulated gene transcripts were common to both affected subjects. Evaluation of the changes in gene expression by functional-enrichment analysis identified several enriched GO pathways, processes, networks, and disease-associated transcripts, some of which are germane to the known functions of GRHL2. The top three upregulated GO pathways were linked to protein-folding maturation, cytoskeleton remodeling, and transcriptional control of lipid biosynthesis (involving genes encoding proopiomelanocortins and mitochondrial enzymes involved in metabolic pathways) (Tables S3–S10). Among the most significantly upregulated GO networks were the signal-transduction pathways and intermediate-filament remodeling (Table S7). Conversely, immune-response signaling; migration-inhibitory-factor-induced cell adhesion, migration, and angiogenesis; and networks of cell-cell and cell-matrix adhesion were downregulated (Table S8).

With regard to skin differentiation and barrier formation, selected alterations in gene expression are presented in Table S11. We also verified potential changes by performing quantitative PCR (qPCR) with RNA from whole skin of three individuals from the two pedigrees, as well as immortalized keratinocytes and primary fibroblasts from one affected person (see Supplemental Data for methods and controls). We observed reduced expression of *GRHL2* for all templates and a contrasting increase in *GRHL1* (MIM 609786) and *GRHL3* (MIM 608317) expression: unique and cooperative roles for this transcription factor family have been previously documented.[24] The most marked skin-barrier-associated gene changes were upregulation of aquaporin-encoding genes *AQP5* (MIM

600442) and *AQP7* (MIM 602974), the latter of which was expressed 50×–100× more in affected skin than in control skin. Gain-of-function mutations in *AQP5* have previously been associated with a form of autosomal-dominant nonepidermolytic palmoplantar keratoderma (MIM 600962).[25] Two-fold or greater reduction in gene expression was noted for *S100A8* (MIM 123885) and *S100A9* (MIM 123886), known targets for *GRHL1*. Previously, it has also been shown that GRHL2 enhances skin-barrier function by upregulating the tight-junction components claudins 3 and 4 and also Rab25, which localizes claudin 4 to tight junctions.[26] In affected people, we noted increases in *CLDN3* (MIM 602910), *CLDN4* (MIM 602909), and *RAB25* (MIM 612942) expression in whole skin (transcriptome and qPCR) and cultured keratinocytes (qPCR). Increased claudin 4 immunolabeling was also noted in the skin of two affected individuals (Figure S5).

Labeling for the proliferation marker Ki-67 was increased in the affected subjects' skin (Figure S4). This indicates that suprabasal keratinocytes are subject to an abnormal terminal-differentiation program, which provides a possible explanation for thickening of the epidermis and impairment of the epidermal barrier in our affected individuals with mutant *GRHL2*, although it is unclear why the most prominent skin scaling was found around the margins of the soles. We also noted reduced expression of *TERT* in the affected individuals' skin and keratinocytes. Overexpression of *GRHL2* in normal keratinocytes increases telomerase activity and increases replicative life span (*TERT* and *PCNA* [MIM 176740]). In contrast, knockdown of *GRHL2* represses the expression of these genes.[12]

The impact of *GRHL2* mutations on cell morphology has been previously described.[4] Lung epithelial cells transduced with *Grhl2* small hairpin RNA flatten in culture and lose their cuboidal morphology into an expanded cell phenotype. Knocking down *Grhl2* in lung epithelial cell lines leads to downregulation of *Cldn4* and *Cdh1*.[9] In our subjects' keratinocytes, immunostaining with E-cadherin showed reduced expression and qPCR showed downregulation of this transcript (Figure S6).

In addition to being expressed in skin, *Grhl2* and *GRHL2* are highly expressed in the inner ear, the lung epithelium, the ureteric bud of the kidney, the olfactory epithelium, the urogenital tract, the gastric mucosa, and human breast cancer cells.[4,8–11,18,27,28] With regard to the clinical phenotype in our affected individuals, aside from the changes affecting the skin and oral mucosa, the other main features comprised deafness and asthma, although this was variably present. Three subjects (ED-01 IV-4 and IV-5 and ED-02 VI-3) had deafness that developed in early infancy (c.f. the later-onset deafness in other families with GRHL2 haploinsufficiency).[15,16] Of note, none of the heterozygous carriers of either missense mutation in *GRHL2* had any deafness. The significance of GRHL2 in vertebrate inner-ear development is well established,[16] but the lack of deafness in the heterozygotes (and some homozygotes) in our pedigrees indicates a different functional effect of the missense mutations. Deafness is not a common feature of ectodermal dysplasia syndromes, although hearing loss can result from abnormalities in p63 and Notch signaling (morphological defects in organ of Corti)[29] and mutations in connexins 26 and 30 (altered endolymph ion homeostasis).[30] In contrast, mutagenesis studies in *Grhl2* have indicated a probable different pathophysiology for deafness with enlarged otocysts, absent otoliths, and malformed semicircular canals.[16]

The observation that three of the affected individuals (ED-01 IV-4 and IV-5 and ED-02 IV-2) had clinical symptoms of asthma is also noteworthy because the top enriched GO disease among the downregulated transcripts in our microarray data was asthma (Table S10). Previous in situ hybridization analyses have indicated that *Grhl2* is the only family member that is highly expressed in distal lung epithelium throughout development, although the particular cells expressing *Grhl2* have not been identified, nor has its functional role in the lung epithelium been fully established.[4] *Grhl1* and *Grhl3*, in contrast, are expressed in the embryonic lung epithelium, but later their expression is reduced in bronchi and bronchioles and is undetectable in the alveolar lung epithelium.[4,27] The potential relevance of other sequence variants in *GRHL2* to sporadic or familial cases of human asthma and other obstructive-airway diseases remains to be determined.

In summary, GRHL2, a member of a family of highly conserved transcription factors, is implicated in epithelial morphogenesis across a number of species. We have used whole-exome sequencing to identify *GRHL2* mutations underlying an ectodermal dysplasia syndrome in two families, and our data expand the current knowledge about the role of *GRHL2* in human disease and epithelial cell biology.

Supplemental Data

Supplemental Data include 6 figures and 13 tables and can be found with this article online at http://dx.doi.org/10.1016/j.ajhg.2014.08.001.

Acknowledgments

The Centre for Dermatology and Genetic Medicine is supported by a Wellcome Trust Strategic Award (reference 098439/Z/12/Z). This work was supported by the Biotechnology and Biological Sciences Research Council, the Royal Society, and the UK National Institute for Health Research comprehensive Biomedical Research Centre award to Guy's and St. Thomas' NHS Foundation Trust in partnership with the King's College London and King's College Hospital NHS Foundation Trust. This study was also supported, in part, by DebRA UK, the Great Britain Sasakawa Foundation (award 4314), and the Strategic Young Researcher Overseas Visits Program for Accelerating Brain Circulation (S2404) from the Japan Society for the Promotion of Science. We also thank Venu Pullabhatla for assistance with transcriptomic data analysis and access.

Received: June 11, 2014
Accepted: August 1, 2014
Published: August 21, 2014

Web Resources

The URLs for data presented herein are as follows:

Ensembl Genome Browser, http://www.ensembl.org/index.html
Gene Expression Omnibus, http://www.ncbi.nlm.nih.gov/geo/
Gene Ontology Consortium, http://www.geneontology.org/
Online Mendelian Inheritance in Man (OMIM), http://www.omim.org/
Primer3, http://frodo.wi.mit.edu/primer3/
PubMed, http://www.ncbi.nlm.nih.gov/PubMed/
RefSeq, http://www.ncbi.nlm.nih.gov/RefSeq
UCSC Genome Browser, http://genome.ucsc.edu/

Accession Numbers

The Gene Expression Omnibus accession number for the microarray data reported in this paper is number GSE56486.

References

1. Venkatesan, K., McManus, H.R., Mello, C.C., Smith, T.F., and Hansen, U. (2003). Functional conservation between members of an ancient duplicated transcription factor family, LSF/Grainyhead. Nucleic Acids Res. *31*, 4304–4316.
2. Stramer, B., and Martin, P. (2005). Cell biology: master regulators of sealing and healing. Curr. Biol. *15*, R425–R427.
3. Werth, M., Walentin, K., Aue, A., Schönheit, J., Wuebken, A., Pode-Shakked, N., Vilianovitch, L., Erdmann, B., Dekel, B., Bader, M., et al. (2010). The transcription factor grainyhead-like 2 regulates the molecular composition of the epithelial apical junctional complex. Development *137*, 3835–3845.
4. Varma, S., Cao, Y., Tagne, J.B., Lakshminarayanan, M., Li, J., Friedman, T.B., Morell, R.J., Warburton, D., Kotton, D.N., and Ramirez, M.I. (2012). The transcription factors Grainyhead-like 2 and NK2-homeobox 1 form a regulatory loop that coordinates lung epithelial cell morphogenesis and differentiation. J. Biol. Chem. *287*, 37282–37295.
5. Bray, S.J., and Kafatos, F.C. (1991). Developmental function of Elf-1: an essential transcription factor during embryogenesis in Drosophila. Genes Dev. *5*, 1672–1683.
6. Narasimha, M., Uv, A., Krejci, A., Brown, N.H., and Bray, S.J. (2008). Grainy head promotes expression of septate junction proteins and influences epithelial morphogenesis. J. Cell Sci. *121*, 747–752.
7. Cieply, B., Farris, J., Denvir, J., Ford, H.L., and Frisch, S.M. (2013). Epithelial-mesenchymal transition and tumor suppression are controlled by a reciprocal feedback loop between ZEB1 and Grainyhead-like-2. Cancer Res. *73*, 6299–6309.
8. Werner, S., Frey, S., Riethdorf, S., Schulze, C., Alawi, M., Kling, L., Vafaizadeh, V., Sauter, G., Terracciano, L., Schumacher, U., et al. (2013). Dual roles of the transcription factor grainyhead-like 2 (GRHL2) in breast cancer. J. Biol. Chem. *288*, 22993–23008.
9. Varma, S., Mahavadi, P., Sasikumar, S., Cushing, L., Hyland, T., Rosser, A.E., Riccardi, D., Lu, J., Kalin, T.V., Kalinichenko, V.V., et al. (2014). Grainyhead-like 2 (GRHL2) distribution reveals novel pathophysiological differences between human idiopathic pulmonary fibrosis and mouse models of pulmonary fibrosis. Am. J. Physiol. Lung Cell. Mol. Physiol. *306*, L405–L419.
10. Mlacki, M., Darido, C., Jane, S.M., and Wilanowski, T. (2014). Loss of Grainy head-like 1 is associated with disruption of the epidermal barrier and squamous cell carcinoma of the skin. PLoS ONE *9*, e89247.
11. Xiang, J., Fu, X., Ran, W., Chen, X., Hang, Z., Mao, H., and Wang, Z. (2013). Expression and role of grainyhead-like 2 in gastric cancer. Med. Oncol. *30*, 714.
12. Chen, W., Xiao Liu, Z., Oh, J.E., Shin, K.H., Kim, R.H., Jiang, M., Park, N.H., and Kang, M.K. (2012). Grainyhead-like 2 (GRHL2) inhibits keratinocyte differentiation through epigenetic mechanism. Cell Death Dis. *3*, e450.
13. Senga, K., Mostov, K.E., Mitaka, T., Miyajima, A., and Tanimizu, N. (2012). Grainyhead-like 2 regulates epithelial morphogenesis by establishing functional tight junctions through the organization of a molecular network among claudin3, claudin4, and Rab25. Mol. Biol. Cell *23*, 2845–2855.
14. Wilanowski, T., Tuckfield, A., Cerruti, L., O'Connell, S., Saint, R., Parekh, V., Tao, J., Cunningham, J.M., and Jane, S.M. (2002). A highly conserved novel family of mammalian developmental transcription factors related to Drosophila grainyhead. Mech. Dev. *114*, 37–50.
15. Peters, L.M., Anderson, D.W., Griffith, A.J., Grundfast, K.M., San Agustin, T.B., Madeo, A.C., Friedman, T.B., and Morell, R.J. (2002). Mutation of a transcription factor, TFCP2L3, causes progressive autosomal dominant hearing loss, DFNA28. Hum. Mol. Genet. *11*, 2877–2885.
16. Vona, B., Nanda, I., Neuner, C., Müller, T., and Haaf, T. (2013). Confirmation of GRHL2 as the gene for the DFNA28 locus. Am. J. Med. Genet. A. *161A*, 2060–2065.
17. Van Laer, L., Van Eyken, E., Fransen, E., Huyghe, J.R., Topsakal, V., Hendrickx, J.J., Hannula, S., Mäki-Torkko, E., Jensen, M., Demeester, K., et al. (2008). The grainyhead like 2 gene (GRHL2), alias TFCP2L3, is associated with age-related hearing impairment. Hum. Mol. Genet. *17*, 159–169.
18. Han, Y., Mu, Y., Li, X., Xu, P., Tong, J., Liu, Z., Ma, T., Zeng, G., Yang, S., Du, J., and Meng, A. (2011). Grhl2 deficiency impairs otic development and hearing ability in a zebrafish model of the progressive dominant hearing loss DFNA28. Hum. Mol. Genet. *20*, 3213–3226.
19. Li, X., Huo, X., Liu, K., Li, X., Wang, M., Chu, H., Hu, F., Sheng, H., Zhang, Z., and Zhu, B. (2013). Association between genetic variations in GRHL2 and noise-induced hearing loss in Chinese high intensity noise exposed workers: a case-control analysis. Ind. Health *51*, 612–621.
20. Rifat, Y., Parekh, V., Wilanowski, T., Hislop, N.R., Auden, A., Ting, S.B., Cunningham, J.M., and Jane, S.M. (2010). Regional neural tube closure defined by the Grainy head-like transcription factors. Dev. Biol. *345*, 237–245.
21. Wilanowski, T., Caddy, J., Ting, S.B., Hislop, N.R., Cerruti, L., Auden, A., Zhao, L.L., Asquith, S., Ellis, S., Sinclair, R., et al. (2008). Perturbed desmosomal cadherin expression in grainy head-like 1-null mice. EMBO J. *27*, 886–897.
22. Ting, S.B., Caddy, J., Hislop, N., Wilanowski, T., Auden, A., Zhao, L.L., Ellis, S., Kaur, P., Uchida, Y., Holleran, W.M., et al. (2005). A homolog of Drosophila grainy head is essential for epidermal integrity in mice. Science *308*, 411–413.
23. Chen, W., Dong, Q., Shin, K.H., Kim, R.H., Oh, J.E., Park, N.H., and Kang, M.K. (2010). Grainyhead-like 2 enhances the human telomerase reverse transcriptase gene expression by inhibiting DNA methylation at the 5′-CpG island in normal human keratinocytes. J. Biol. Chem. *285*, 40852–40863.
24. Boglev, Y., Wilanowski, T., Caddy, J., Parekh, V., Auden, A., Darido, C., Hislop, N.R., Cangkrama, M., Ting, S.B., and Jane, S.M. (2011). The unique and cooperative roles of the Grainy

head-like transcription factors in epidermal development reflect unexpected target gene specificity. Dev. Biol. *349*, 512–522.
25. Blaydon, D.C., Lind, L.K., Plagnol, V., Linton, K.J., Smith, F.J., Wilson, N.J., McLean, W.H., Munro, C.S., South, A.P., Leigh, I.M., et al. (2013). Mutations in AQP5, encoding a water-channel protein, cause autosomal-dominant diffuse nonepidermolytic palmoplantar keratoderma. Am. J. Hum. Genet. *93*, 330–335.
26. Tanimizu, N., and Mitaka, T. (2013). Role of grainyhead-like 2 in the formation of functional tight junctions. Tissue Barriers *1*, e23495.
27. Kang, X., Chen, W., Kim, R.H., Kang, M.K., and Park, N.H. (2009). Regulation of the hTERT promoter activity by MSH2, the hnRNPs K and D, and GRHL2 in human oral squamous cell carcinoma cells. Oncogene *28*, 565–574.
28. Auden, A., Caddy, J., Wilanowski, T., Ting, S.B., Cunningham, J.M., and Jane, S.M. (2006). Spatial and temporal expression of the Grainyhead-like transcription factor family during murine development. Gene Expr. Patterns *6*, 964–970.
29. Terrinoni, A., Serra, V., Bruno, E., Strasser, A., Valente, E., Flores, E.R., van Bokhoven, H., Lu, X., Knight, R.A., and Melino, G. (2013). Role of p63 and the Notch pathway in cochlea development and sensorineural deafness. Proc. Natl. Acad. Sci. USA *110*, 7300–7305.
30. Terrinoni, A., Codispoti, A., Serra, V., Bruno, E., Didona, B., Paradisi, M., Nisticò, S., Campione, E., Napolitano, B., Diluvio, L., and Melino, G. (2010). Connexin 26 (GJB2) mutations as a cause of the KID syndrome with hearing loss. Biochem. Biophys. Res. Commun. *395*, 25–30.

The American Journal of Human Genetics, Volume *95*

Supplemental Data

Mutations in *GRHL2* Result in an Autosomal-Recessive Ectodermal Dysplasia Syndrome

Gabriela Petrof, Arti Nanda, Jake Howden, Takuya Takeichi, James R. McMillan, Sophia Aristodemou, Linda Ozoemena, Lu Liu, Andrew P. South, Celine Pourreyron, Dimitra Dafou, Laura E. Proudfoot, Hejab Al-Ajmi, Masashi Akiyama, W.H. Irwin McLean, Michael A. Simpson, Maddy Parsons, and John A. McGrath

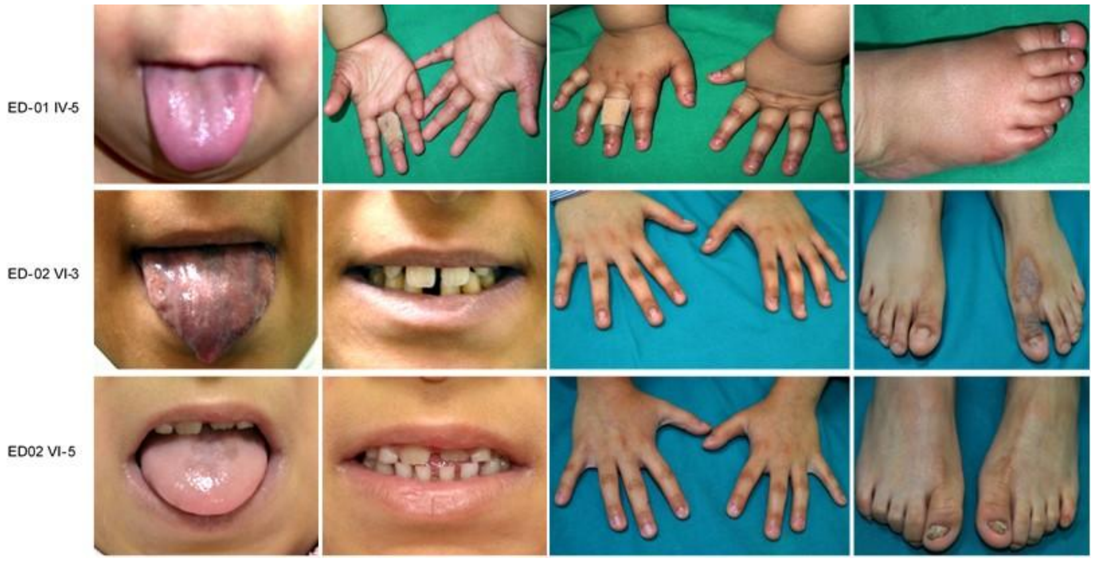

Figure S1. Clinical illustration of this ectodermal dysplasia syndrome. Additional clinical images of other affected members from both pedigrees (See also Figure 1 in main manuscript).

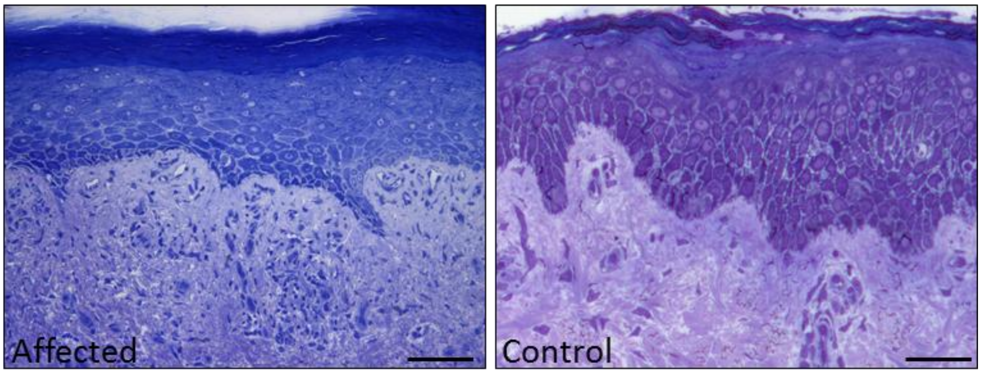

Figure S2. Skin histology. Light microscopy of skin sample from the foot of ED-01 IV-4 shows mild acanthosis and hyperkeratosis (Richardson's stain; scale bar represents 50µm).

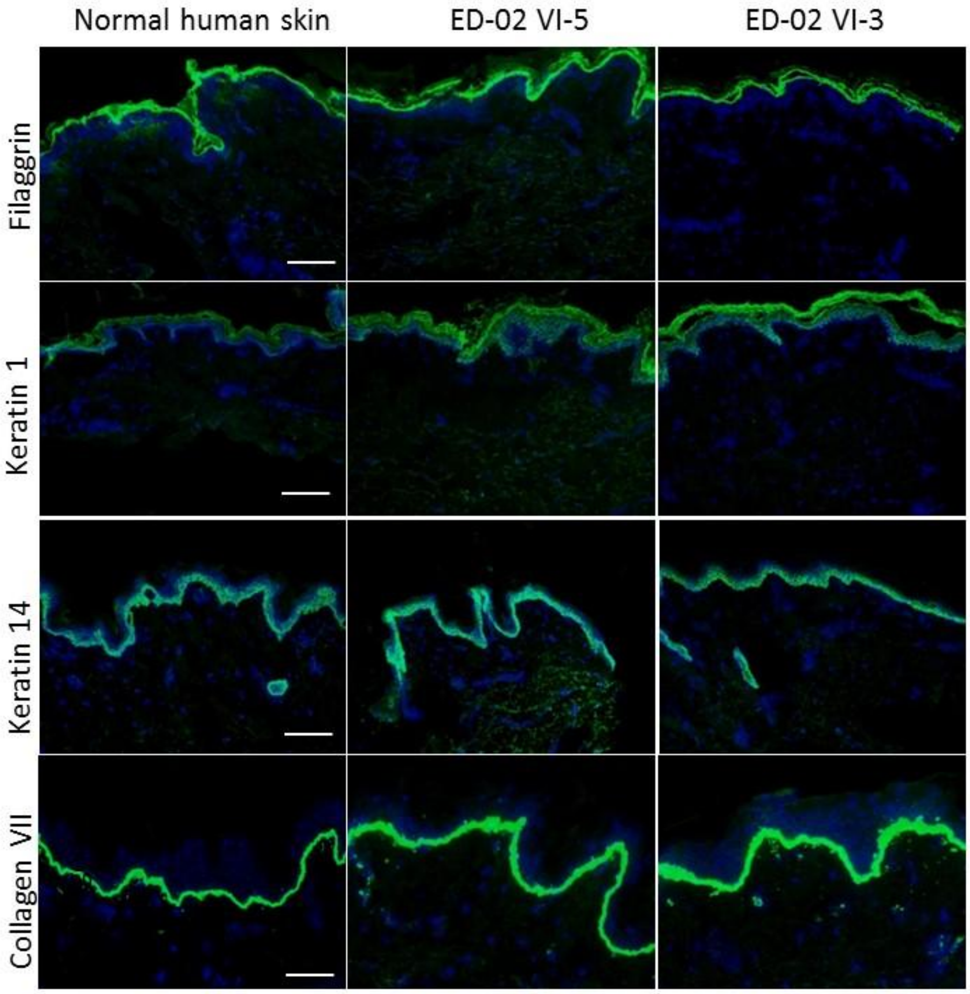

Figure S3. Skin immunolabeling. Immunofluorescence microscopy reveals no major differences in intensity labeling for several epidermal and basement membrane proteins in 2 affected individuals in pedigree ED-02 (right panels) compared to control skin (left panel). Scale bars represent 50 µm. (See Table S13 for antibody details).

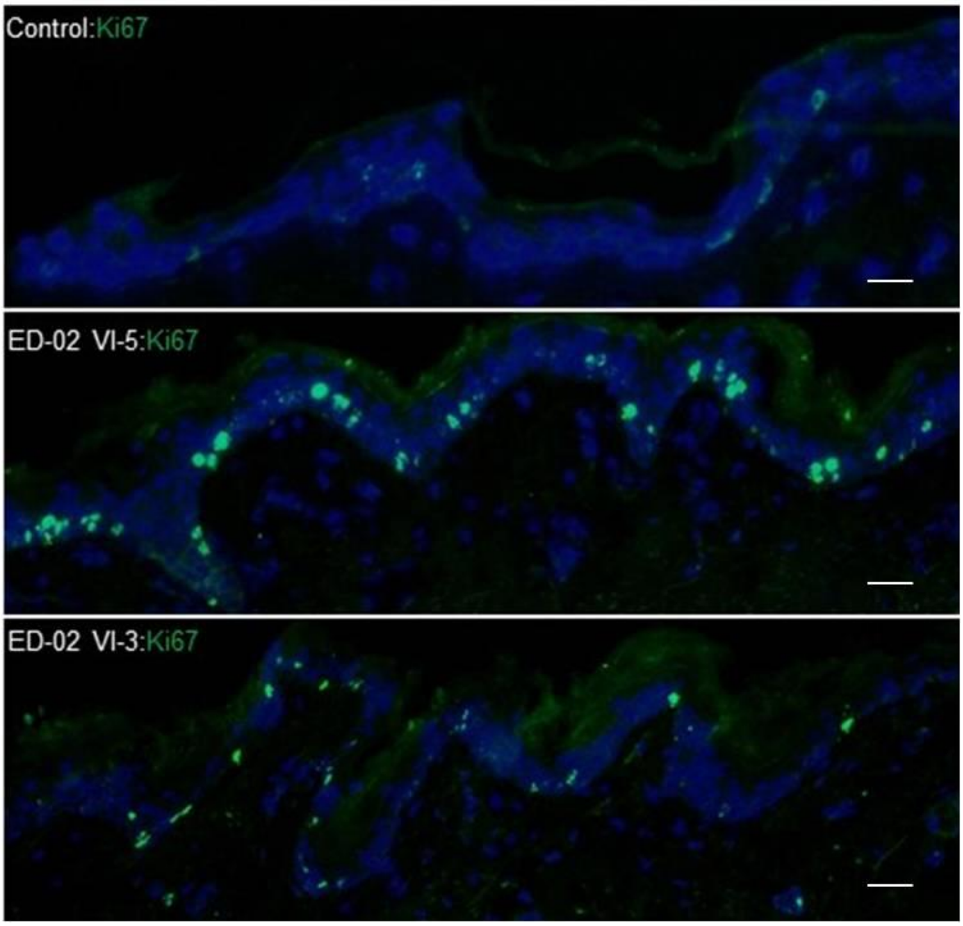

Figure S4. Skin immunolabeling for Ki67. Representative images of control and affected person skin sections stained for the proliferation marker Ki67. Ki-67 labeling is increased in the epidermis of affected individuals. Scale bars represent 50 µm.

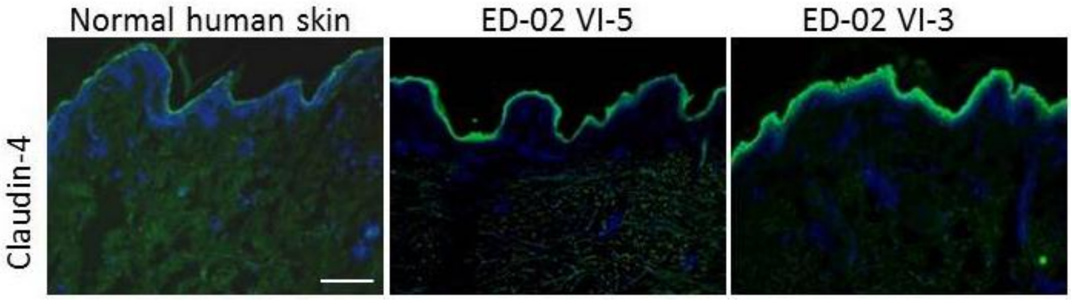

Figure S5. Skin immunolabeling for claudin-4. Immunofluorescence microscopy reveals increased intensity labeling for claudin-4 in two affected individuals in pedigree ED-02 (right panels) compared to control skin (left panel). Scale bars represent 50 µm.

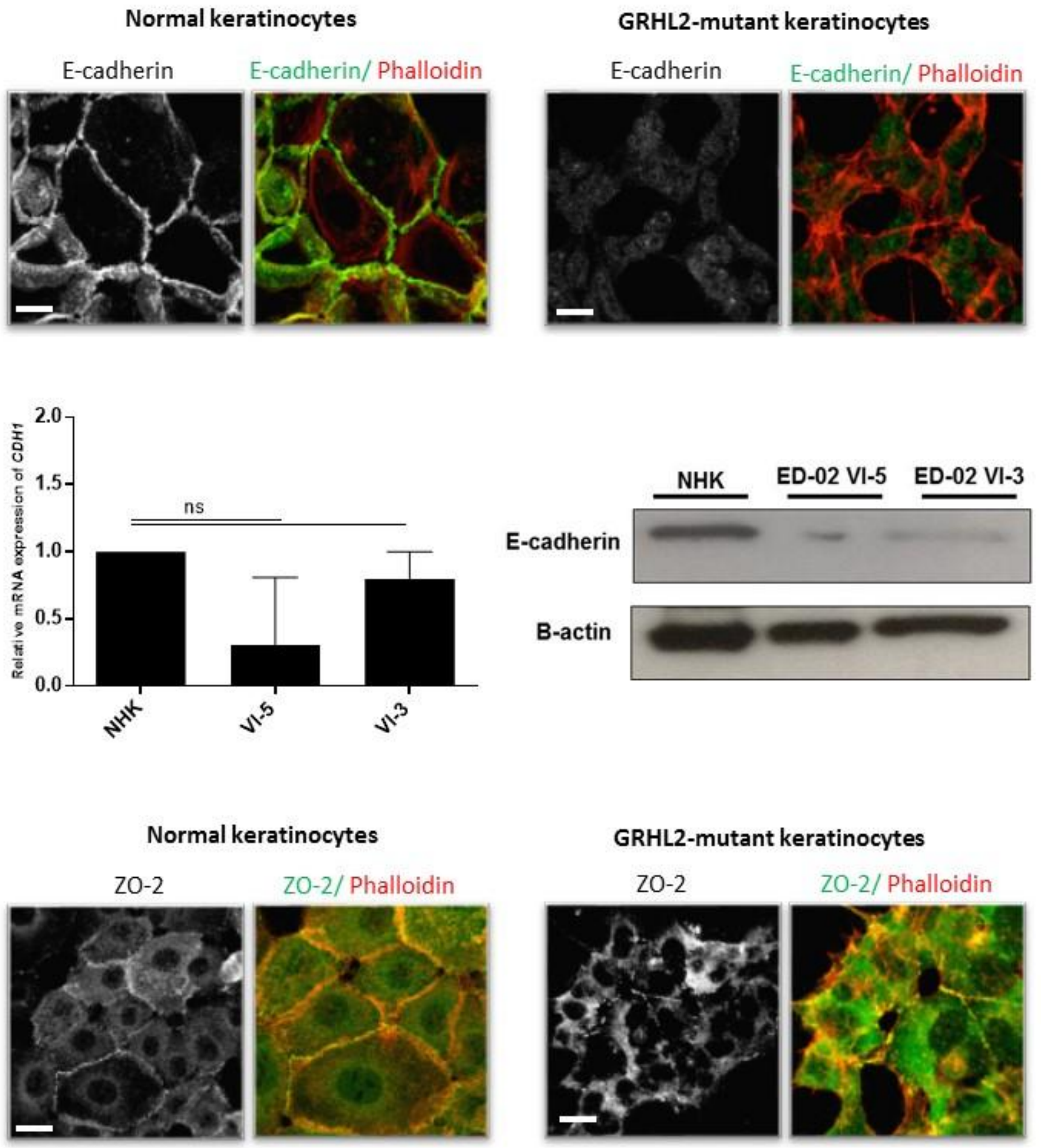

Figure S6. Impact of mutations in *GRHL2* on E-cadherin and zona occludens-2. In cultured keratinocytes staining for the adherens junctional protein E-cadherin appears reduced in the mutant cells with less cell membrane labeling. E-cadherin is reduced at a protein level (Western blotting) although gene expression is not significantly reduced by qPCR. The membranous localization of the tight junction protein zona occludens-2 (ZO-2) is also diminished in mutant keratinocytes. Phalloidin labels the actin cytoskeleton. Scale bar represents 20 µm.

TABLE S1: EXOME SEQUENCING DETAILS

**(Patient ED-01 IV-4)**	
Total sequence reads	18908110
Quality control passed aligned reads	14667221
%	77.57
Quality control passed aligned reads +/- 150bp	17112569
%	90.50
Mean read depth	26.80
% of exome > 1x reads	96.82
% of exome > 5x reads	89.51
% of exome >10x reads	76.05
% of exome >20x reads	49.10
**(Patient ED-02 VI-3)**	
Total sequence reads	79571546
Quality control passed aligned reads	56980960
%	71.61
Quality control passed aligned reads +/- 150bp	64235288
%	80.73
Mean read depth	105.46
% of exome > 1x reads	98.12
% of exome > 5x reads	96.44
% of exome >10x reads	94.64
% of exome >20x reads	89.92

TABLE S2. FILTERED EXOME VARIANTS

Patient ED-01 IV:4					
Gene	Gene name	Mutation	Size of homozygous block (Mb)	SIFT	Poly-phen2
CLCNKA	Chloride channel protein ClC-Ka isoform 2	Splicing	0.834	-	-
GDAP1	Ganglioside-induced differentiation-associated	Nonsynonymous SNV	56.9	Damaging (0.03)	Benign (0.139)
GRHL2	Grainyhead-like protein 2 homolog	Nonsynonymous SNV	56.9	Tolerated (0.52)	Probably damaging (0.994)
RRM2B	Gibonucleoside-diphosphate reductase subunit M2	Nonsynonymous SNV	56.9	Tolerated (0.07)	Benign (0)
CAND1	Cullin-associated and neddylation	Splicing	12.5	-	-
MDM1	Nuclear protein MDM1	Splicing	12.5	-	-
NIN	Ninein isoform 1	Nonsynonymous SNV	14.8	Damaging (0.05)	Probably damaging (0.961)
GOLGA6L6	Golgin A6 family-like 6	Nonsynonymous SNV	0.000282	Tolerated (0.08)	Probably damaging (0.998)
APOL3	Apolipoprotein L, 3	Nonsynonymous SNV	19.5	Tolerated (0.07)	Probably damaging (0.993)
MKL1	MKL (megakaryoblastic leukemia)/myocardin-like	Nonsynonymous SNV	19.5	Damaging (0.05)	Probably damaging (1)
**Patient ED-02 VI:3**					
**Gene**	**Gene name**	**Mutation**	**Size of homozygous block (Mb)**	**SIFT**	**Poly-phen2**
PROM1	Prominin 1	Nonsynonymous SNV	1.88	Tolerated (0.54)	Possibly damaging (0.513)

TBC1D1	TBC1 (tre-2/USP6, BUB2, cdc16) domain family, member 1	Splicing	0.0464	-	-
RAB11FIP1	RAB11 family interacting protein 1 (class I)	Nonsynonymous SNV	21.8	Tolerated (0.36)	Benign (0.001)
GGH	Gamma-glutamyl hydrolase (conjugase, folylpolygammaglutamyl hydrolase)	Nonsynonymous SNV	36.6	Tolerated (0.11)	Probably damaging (0.998)
GRHL2	Grainyhead-like protein 2 homolog	Nonsynonymous SNV	19.6	Tolerated (0.28)	Probably damaging (0.984)
DUSP14	Dual specificity phosphatase 14	splicing	0.153	-	-

Legend: Hom: homozygous, SNV: single nucleotide variation.

This table shows the size of the homozygous blocks of homozygosity surrounding each novel homozygous mutation and the predicted pathogenicity of the missense mutations. The sizes of the putative regions of homozygosity were estimated by the distance between the delimiting heterozygous variants identified by whole-exome sequencing. The SIFT score ranges from 0 to 1. Values above 0.05 are predicted to be tolerated and values below 0.05 are considered damaging. The Polyphen-2 scoring system differs with values greater than 0.5 regarded as possibly damaging and below 0.5 reported as benign. SNV: single nucleotide variation.

Table S3. Enriched GO Pathways amongst up-regulated transcripts.

#	Pathways	p-Value
**1**	Protein folding and maturation_POMC processing	7.53e-22
**2**	SCAP/SREBP transcriptional control of cholesterol and FA biosynthesis	1.653-09
**3**	Regulation of metabolism_Bile acid regulation of glucose and lipid metabolism via FXR	4.40e-06
**4**	Role of diethylhexyl Phthalate and Tributylin in fat cell diffentiation	8.14e-06
**5**	Cytoskeleton remodeling_Keratin filaments	3.71e-05
**6**	Cell cycle_Spindle assembly and chromosome separation	2.01e-04
**7**	Phenylalanine metabolism/Rodent version	3.37e-04
**8**	Phenylalanine metabolism	3.74e-04
**9**	Pyruvate metabolism	4.37e-04
**10**	Cell adhesion_tight junctions	7.40e-04

N.B. Protein folding and maturation_POMC processing was enriched in several other unrelated experiments on the microarray and may be a spurious finding.

Table S4. **Enriched GO Pathways amongst down-regulated transcripts.**

#	Pathways	p-Value
**1**	Immune response_HSP60 and HSP70/TLR signaling pathway	7.57e-08
**2**	Immune response_MIF-induced cell adhesion, migration and angiogenesis	1.30e-07
**3**	Reproduction_GnRH signaling	7.49e-07
**4**	Immune response IL-5 signaling	1.15e-06
**5**	Stellate cells activation and liver fibrosis	1.39e-06
**6**	Immune response_IL-18 signaling	1.40e-06
**7**	Immune response_Immunological synapse formation	1.81e-06
**8**	Immune response_C5a signaling	3.34e-06
**9**	Development_GM-CSF signaling	3.58e-06
**10**	Immune response_Oncostatin M signaling via MAPK in human cells	4.36e-06

Table S5. Enriched GO Processes amongst up-regulated transcripts.

#	Processes	p-Value
**1**	Lipid metabolic process	7.01e-23
**2**	Small molecule metabolic process	2.97e-17
**3**	Cellular lipid metabolic process	4.53e-12
**4**	Fatty acid metabolic process	1.52e-11
**5**	Metabolic process	9.03e-10
**6**	Ferric iron transport	5.51e-09
**7**	Fatty acid biosynthetic process	7.41e-09
**8**	Triglyceride biosynthetic process	9.95e-08
**9**	Cellular response to cAMP	7.95e-07
**10**	Response to lithium ion	8.22e-07

Table S6. Enriched GO Processes amongst down-regulated transcripts.

#	Processes	p-Value
**1**	Cell adhesion	3.77e-17
**2**	Extracellular matrix organization	4.81e-17
**3**	Inflammatory response	4.54e-16
**4**	Innate immune response	2.52e-15
**5**	Immune response	1.25e-14
**6**	Response to estradiol stimulus	2.78e-14
**7**	Response to lipopolysaccharide	7.46e-14
**8**	Neutrophil chemotaxis	3.31e-13
**9**	Interferon-gamma-mediated signaling pathway	4.79e-13
**10**	Response to mechanical stimulus	7.05e-12

Table S7. Enriched GO Networks amongst up-regulated transcripts.

#	Networks	p-Value
**1**	Signal transduction_Neuropeptide signaling pathways	1.54e-08
**2**	Cytoskeleton_Intermediate filaments	2.05e-05
**3**	Reproduction_Progesterone signaling	4.79e-05
**4**	Cell adhesion_Cell junctions	8.09e-05
**5**	Signal transduction_WNT signaling	2.18e-04
**6**	Reproduction_Spermatogenesis, motility and copulation	4.09e-04
**7**	Cytoskeleton_Actin filaments	2.05e-03
**8**	Regulation of metabolism_Bile acid regulation of lipid metabolism and negative FXR-dependent regulation of bile acids concentration	3.10e-03
**9**	Transport_Iron transport	4.49e-03
**10**	Proteolysis_Connective tissue degradation	8.42e-03

Table S8. Enriched GO Networks amongst down-regulated transcripts.

#	Networks	p-Value
**1**	Cell adhesion_Cell matrix interactions	6.79e-13
**2**	Cell adhesion_Platelet-endothelium-leucocyte interactions	3.71e-11
**3**	Immune response_Antigen presentation	2.84e-06
**4**	Immune response_Th17-derived cytokines	5.10e-06
**5**	Inflammation_IL-4 signaling	1.56e-05
**6**	Development_Regulation of angiogenesis	6.89e-05
**7**	Development_EMT_Regulation of epithelial-to-mesenchymal transition	8.99e-05
**8**	Cell adhesion_Integrin-mediated cell-matrix adhesion	1.05e-04
**9**	Immune response_IL-5 signaling	1.32e-04
**10**	Reproduction_Gonadotropin regulation	1.43e-04

Table S9. Enriched GO Diseases amongst up-regulated transcripts.

#	Diseases	p-Value
**1**	Adrenal insufficiency	2.01e-26
**2**	Cushing syndrome	9.46e-21
**3**	Pain	4.53e-19
**4**	Eclampsia	6.74e-19
**5**	Hepatitis, autoimmune	2.28e-18
**6**	Fatigue Syndrome, Chronic	3.07e-17
**7**	Osteoporosis	1.08e-17
**8**	Mesothelioma	1.49e-17
**9**	Obesity, Morbid	3.16e-17
**10**	Depressive disorder	1.31e-16

Table S10. Enriched GO Diseases amongst down-regulated transcripts.

#	Diseases	p-Value
**1**	Asthma	2.28e-28
**2**	Arthritis, Rheumatoid	1.60e-26
**3**	Wounds and Injuries	1.04e-25
**4**	Inflammation	1.06e-22
**5**	Fibrosis	2.32e-21
**6**	Arthritis	1.54e-20
**7**	Pulmonary disease, chronic obstructive	2.06e-19
**8**	Colitis, ulcerative	2.12e-18
**9**	Melanoma, cutaneous malignant	3.47e-18
**10**	Carcinoma, non-small cell lung	4.36e-18

Table S11. **Skin differentiation/barrier gene expression levels.** Gene expression levels were compared using RNA from whole skin of 3 individuals from the two pedigrees, as well as immortalized keratinocytes and primary fibroblasts from one affected subject (ED-02 VI-5) relative to control samples. ND = Not Detected.

Gene symbol	Transcriptomic data (fold change)		Qualitative Real-time PCR				
	ED-02 VI-5 whole skin	ED-02 VI-3 Whole skin	ED-02 VI-5 Immortalized keratinocytes	ED-02 VI-5 Primary fibroblasts	ED-01 IV-4 Whole skin	ED-02 VI-5 Whole skin	ED-02 VI-3 Whole skin
GRHL1	1.2	1.4	1.64	ND	1.17	8.3	2.76
GRHL2	0.78	0.66	0.2	ND	0.37	0.76	0.37
GRHL3	1.16	1.39	1.52	ND	1.87	2.73	4.06
CDH1	1.17	1.1	0.5	ND	0.44	1.19	3.2
KRT10	0.89	0.91	0.25	ND	0.99	0.79	0.89
KRT14	1.21	1.15	3.2	ND	1.23	2.43	2.85
S100A8	0.38	0.22	0.50	ND	0.59	0.12	0.48
IVL	1.65	1.42	0.24	ND	0.70	13.82	3.73
FLG	1.05	1.13	0.28	ND	0.05	11.65	4.86
CLDN3	1.47	2.38	4.70	ND	19.27	11.71	8.33
CLDN4	1.8	1.94	1.87	ND	1.61	16.07	3.45
RAB25	1.38	1.47	2.79	0.14	1.14	1.81	2.85
TSLP	0.43	1.82	0.61	0.34	0.62	0.75	1.15
DSG1	1.1	1.13	9.41	ND	0.71	1.42	3.58
TJP2	1.32	1.1	ND	1.0	1.13	1.05	1.31
TERT	0.84	0.68	0.66	ND	ND	0.21	0.12
AQP7	52.7	119.5	8.24	ND	5.35	81.6	105.36
TGM1	1.0	1.38	0.03	ND	0.01	0.95	1.45

These data allow for verification of transcriptomic data by qPCR, confirmation of gene expression changes for different mutations in *GRHL2*, and insight into gene expression similarities and differences in pure cell populations (keratinocytes/fibroblasts) and well as in *in vivo* differentiated skin. The homozygous missense mutations in *GRHL2* are associated with upregulation of *GRHL1*, *GRHL3*, *KRT14* (keratin 14), *CLDN3* (claudin 3), *CLDN4* (claudin 4), *RAB25* (Ras-related protein Rab-25) and *AQP7* (aquaporin 7). The mutations also are found in association with reduced expression of *GRHL2*, *KRT10* (keratin 10), and *TERT* (telomerase reverse transcriptase). Inter-individual differences were seen for *CDH1* (E-cadherin), *IVL* (involucrin), *FLG* (profilaggrin), *TSLP* (thymic stromal lymphopoietin), *DSG1* (desmoglein1) and *TGM1* (transglutaminase 1).

Table S12. List of Taqman Gene expression assays used for qPCR. Following the extraction and quantification procedure, cDNA was produced by reverse transcription (RT) of RNA using the High Capacity Reverse Transcription kit (Applied Biosystems) according to the manufacturer's instructions. TaqMan_ Gene Expression Assays were purchased from Applied Biosystems (Warrington, U.K.) and used as directed. Samples were run and analyzed using the 7000HT Real-Time PCR System (Applied Biosystems). Expression values for each primer set were normalized to 18S ribosomal RNA (rRNA) gene values. The relative expression in the affected people samples vs. respective control samples was then determined using the DDCt method.

Gene symbol	Taqman Gene expression Assay
***GRHL1* [MIM 609786]**	Hs01119372_m1
***GRHL2* [MIM 609576]**	Hs00227745_m1
***GRHL3* [MIM 609317]**	Hs00297962_m1

***FLG* [MIM 135490]**	Hs00856927_g1
***IVL* [MIM 147360]**	Hs00846307_s1
***CDH1* [MIM 192090]**	Hs01023894_m1
***KRT10* [MIM 148080]**	Hs00166289_m1
***KRT14* [MIM 148066]**	Hs00265033_m1
***S100A8* [MIM 123885]**	Hs00374264_g1
***CLDN3* [MIM 602910]**	Hs00265816_s1
***CLDN4* [MIM 602909]**	Hs00976831_s1
***RAB25* [MIM 612942]**	Hs01040784_m1
***TSLP* [MIM 607003]**	Hs00263639_m1
***DSG1* [MIM 125670]**	Hs00355084_m1
***TJP2* [MIM 607709]**	Hs00910543_m1
***TERT* [MIM 187270]**	Hs00972656_m1
***AQP7* [MIM 602974]**	Hs00357359_m1
***TGM1* [MIM 190195]**	Hs01070310_m1

Table S13: Antibodies used in this study

rabbit anti-GRHL2	1:500	(HPA004820, Sigma-Aldrich, UK)
mouse anti-claudin-4	1:200	(3E2C1, Life Technologies, Paisley, UK)
mouse anti-collagen VII	1:150	(C6805, Sigma-Aldrich)
mouse anti-E-cadherin	1:1,000	(HECD1, Life Technologies)
mouse anti-keratin 14	1:100	(LL02, Bio-Rad AbD Serotec, San Francisco, CA)
rabbit anti-keratin 5	1:100	(ab24647, Abcam, Cambridge, UK)
mouse anti-cytokeratin 1	1:20	(34Bb4, Enzo Diagnostics, New York, NY)
mouse anti-keratin 10	1:20	(LHP1, Abnova)
mouse anti-filaggrin	1:20	(FLG01, Abcam)
mouse anti-involucrin	1:20	(SY5, Santa Cruz Biotechnology, Santa Cruz, CA)
rabbit anti-ki67	1:500	(ab15580, Abcam)
mouse β-actin	1:10,000	(AC-74, Abcam)
phalloidin-Alexa-568	1:400	(Life Technologies).
